# Transcriptomic and functional analysis of the oosome, a unique form of germ plasm in the wasp *Nasonia vitripennis*

**DOI:** 10.1186/s12915-019-0696-7

**Published:** 2019-10-10

**Authors:** Honghu Quan, Deanna Arsala, Jeremy A. Lynch

**Affiliations:** 1 Department of Pathology and Department of Cell and Molecular Biology, St. Jude Children’s Research Hospital, Memphis, TN 38105 USA; 2 Department of Biological Sciences, University of Illinois at Chicago, Chicago, IL 60607 USA

**Keywords:** Oosome, Germ plasm, Primordial germ cells, Germline, Nasonia, Drosophila, RNAseq, RNA interference

## Abstract

**Background:**

The oosome is the germline determinant in the wasp *Nasonia vitripennis* and is homologous to the polar granules of *Drosophila*. Despite a common evolutionary origin and developmental role, the oosome is morphologically quite distinct from polar granules. It is a solid sphere that migrates within the cytoplasm before budding out and forming pole cells.

**Results:**

To gain an understanding of both the molecular basis of oosome development and the conserved essential features of germ plasm, we quantified and compared transcript levels between embryo fragments that contained the oosome and those that did not. The identity of the differentially localized transcripts indicated that *Nasonia* uses a distinct set of molecules to carry out conserved germ plasm functions. In addition, functional testing of a sample of localized transcripts revealed potentially novel mechanisms of ribonucleoprotein assembly and pole cell cellularization in the wasp.

**Conclusions:**

Our results demonstrate that the composition of germ plasm varies significantly within Holometabola, as very few mRNAs share localization to the oosome and polar granules. Some of this variability appears to be related to the unique properties of the oosome relative to the polar granules in *Drosophila*, and some may be related to differences in pole formation between species. This work will serve as the basis for further investigation into the patterns of germline determinant evolution among insects, the molecular basis of the unique properties of the oosome, and the incorporation of novel components into developmental networks.

**Electronic supplementary material:**

The online version of this article (10.1186/s12915-019-0696-7) contains supplementary material, which is available to authorized users.

## Background

The germline is tasked with producing the gametes, which are essential for the production of a new generation of progeny in sexually reproducing organism. While the requirement for a germline is nearly universal in complex multicellular organisms, the mechanisms used to specify it vary greatly [1]. There are two major strategies for germline specification among animals: zygotic induction and maternal provision. In zygotic induction, inductive signals from surrounding tissues drive the establishment of germline fate, usually relatively late in embryogenesis, after the transition from maternal to zygotic control [1]. In contrast, in the maternal provision mode, the germ cells are specified by determinants synthesized and deposited during oogenesis. This substance, termed germ plasm, has been shown to be both necessary and sufficient to establish the germline fate [2–4]. Primordial germ cells are usually the first cell fate specified in organisms with maternal provision of germ plasm, and specification in this mode occurs prior to the activation of the zygotic genome [1, 5].

It is likely that the maternal provision mode of germ plasm evolved multiple times among the animals, and this is reflected in the highly variable, and often novel, molecular basis of germ plasm determinants across animals [6]. For example, germ plasm in vertebrates (where it exists) is dependent on the maternal localization of *bucky ball* [7] a novel gene found only in vertebrates without clear homologs in other lineages [8]. Similarly, the gene products of *oskar* (*osk*), another novel gene that appears to have originated as a chimera between a LOTUS domain-containing gene and a bacterial lipase [9, 10], are both necessary and sufficient to induce germ plasm and, thus, primordial germ cells (PGCs) in the fly *Drosophila* [11–14]. Downstream of these nucleators is a suite of highly conserved germline-associated molecules (i.e., Vasa (Vas), Nanos (Nos), Tudor (Tud)) that are recruited to the germ plasm, where the nucleators are active [9, 12, 15, 16].

There are several conserved properties of PGCs that are downstream of the initiation of the germline specification cascade. Some of these may be directly encoded in the germ plasm. One common feature is a period of transcriptional quiescence germ cells undergo after being specified [17]. In *Drosophila*, quiescence is mediated by the products of *nos* and *polar granule component* (*pgc*), which are localized to the germ plasm [18–20]. If very early quiescence is broadly conserved, the composition of a species’ germ plasm is especially important since germline-specific developmental events prior to re-initiation of transcription will depend entirely on macromolecules supplied by the germ plasm.

Another broadly conserved property of germ cells is that they undertake significant migration in many species as they seek to colonize the developing gonad [21]. In addition, they are often highly enriched for mitochondria and have specific metabolic needs [22]. Since they carry the genome that will be passed to future generations, germ cells have enhanced mechanisms to prevent DNA damage and to reduce the activity of transposable elements [23, 24].

Which essential features of the PGCs are encoded directly in the germ plasm and how variable the function of this organelle is across species are still open questions since germ plasm has only been looked at in great detail in a very few species. We propose that there is likely to be variation in the details of these conserved features of germline determination, whether due to selective or neutral forces. Pressures that could impact the composition of the germ plasm include as follows: differential activity of transposable elements in the genome, or a novel path for migration of the PGCs to the gonad, for example. Novelties in embryogenesis may also drive germ plasm composition. For example, in several lineages of Diptera, chromosomes are eliminated from somatic nuclei in early cleavage stages, while they are maintained in pole cells [25–27]. Elegant experiments showed that when the germ plasm was destroyed, or displaced from early cleavage nuclei by centrifugation, chromosome elimination proceeded in all nuclei [25, 26]. These results strongly suggested that molecules localized to the germ plasm have a specific role in preventing chromosome elimination in the presumptive germline [26].

**Fig. 1:**
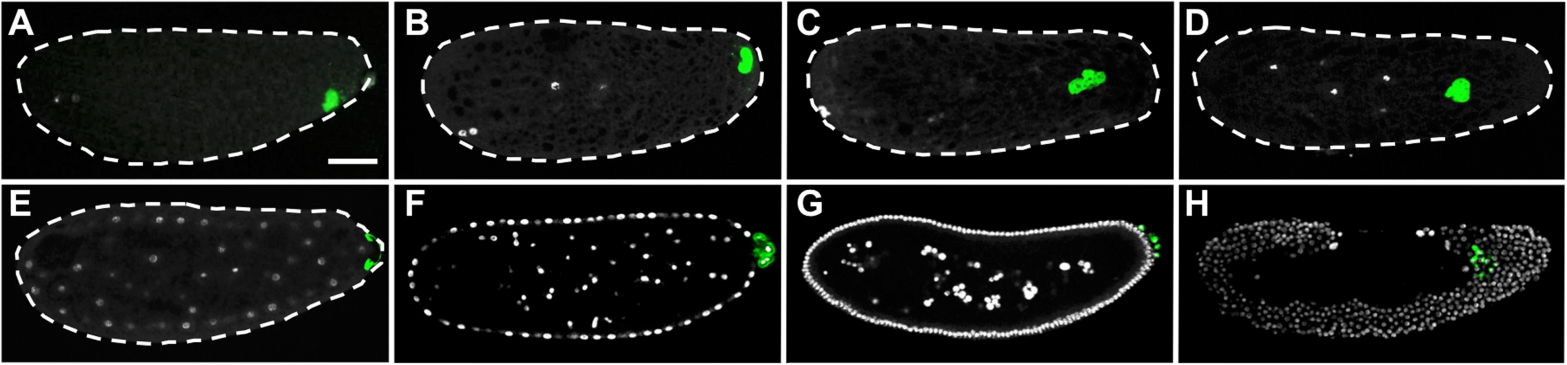
The behavior and fate of the oosome. Green signal produced by fluorescent in situ hybridization (FISH) against *Nv-bark*, white is DAPI staining of DNA. The white dashed lines mark the edges of the embryos where it is not clear. Anterior is at left, and dorsal is up in all images. **a–d** Pre-blastoderm stage: The oosome changes shape and position within the embryo prior blastoderm formation. **e** Early blastoderm stage: The oosome flattens into the bud (green) as the nuclei reach the embryonic cortex. **f** Mid-blastoderm stage: Several pole cells immediately form from the bud in **e**, and remain in a separate cluster on top of the syncytial blastoderm directly at the posterior pole. **g** Late blastoderm stage: The pole cells divide several times to make a complex cluster of cells. **h** Post-gastrulation: Primordial germ cells form clusters in the interior of the embryo. Scale bar indicates 50 μm

Holometabolous insects are an ideal system with which to study how germ plasm evolves, given the unparalleled levels of diversity found among these insects, and the strong baseline understanding of the holometabolan type of germ plasm obtained by research in *Drosophila melanogaster*. Here, we focus on the parasitic wasp *Nasonia vitripennis* as a model to compare to the fruit fly. Like *Drosophila*, *Nasonia* depends on Osk, Vas, and Tud to assemble the germ plasm [9, 28]. However, in contrast to the collection of small granules stably associated with the posterior pole that make up the *Drosophila* germ plasm, the *Nasonia* germ plasm forms a very large, dense organelle called the oosome (Fig. 1). This highly divergent morphology strongly suggests that the composition of the *Nasonia* oosome may be significantly different from the polar granules of *Drosophila*.

The behaviors of the oosome and the PGCs in *Nasonia* further imply a divergent composition of the oosome. In freshly laid eggs, the oosome is tightly bound to the ventral-posterior cortex of the embryo (Fig. 1a). When the zygotic nucleus forms and moves into the interior of the embryo, the oosome detaches from the cortex and coalesces into discrete, nearly spherical structure in the same central column of the cytoplasm as the syncytial nuclei (note that the oosome is in the same focal planes as the pre-blastoderm nuclei during the first five nuclear cycles). It migrates anteriorly in the central column of cytoplasm, before migrating back to the posterior pole (Fig. 1b–d). As the cleavage nuclei migrate toward the cortex, the oosome flattens into a crescent on the posterior pole of the embryo while a large bud protrudes from the pole (Fig. 1e). Typically, two or three nuclei become associated with the bud and the oosome material. The bud pinches off, and the nuclei rapidly individuate into pole cells (Fig. 1f, g). This is distinct from pole cell formation in *Drosophila*, where each pole cell forms individually [29].

In *Nasonia*, the initial pole bud divides to form a cluster of several cells that remains at the posterior pole of the egg until the onset of mesoderm internalization. At this point, the pole cells migrate through the posterior blastoderm epithelium [30] and coalesce into two masses, presumably where the primordial gonads are developing (Fig. 1h). This migration is much earlier and distinct from *Drosophila* pole cells which migrate through the wall of the posterior midgut, well after internalization of the mesoderm and germ band extension have been completed [29].

Thus, it is clear that *Nasonia* and *Drosophila* share some fundamental aspects of germline establishment, but they also have their own diverged features. This raises the question of which genes are the core components for the maternal provision mode and which genes contribute to their own distinct features in germline development.

To address these questions, we compared the mRNA content of anterior and posterior halves of the pre-blastoderm-stage *Nasonia* embryos in an effort to identify the components specifically localized to the oosome. We found only a few mRNAs with conserved localization in both fly polar granules and the *Nasonia* oosome, such as *osk*, *nos*, and *ovo*. The rest either lack *Drosophila* homologs or have homologs in *Drosophila* that do not play any roles in germline development. We performed functional studies for a set of localized transcripts, all of which showed roles in either oosome stability and/or pole cell formation, demonstrating the value of our approach to identify the molecular sources of novelty among various insect lineages.

## Results

### RNAseq analyses of the anterior and posterior poles of the wasp *Nasonia* early embryos

To identify the maternal transcripts in the oosome, we isolated the total RNA separately from anterior and posterior halves of 0–2-h-old (prior to pole cell formation, which occurs at ~ 3 h after egg lay at 25 °C [31]) *Nasonia vitripennis* embryos using the “embryo guillotine” apparatus described by Ding and Lipshitz [32]. A variety of library preparation and sequence analysis approaches (see “Methods”) were used to identify 90 transcripts with statistically significant enrichment at the posterior pole of the *Nasonia*. These ranged in levels of enrichment from 1.3 to 55 times higher in the posterior fragments compared to anterior fragments [33]. Our analyses also uncovered 87 mRNAs significantly enriched at the anterior, with a range of fold enrichment from 1.3 to 9.4 times higher at the anterior [33].

The goal of the work presented here was to identify all transcripts enriched in the oosome, and we believe we reached the maximum utilization of the raw data we obtained from both experiments by varying different analytical conditions. However, no approach can guarantee comprehensiveness, and some molecules may have been missed for several reasons. These include, for example, the difficulty in detecting differential splice-form localization, the potential incompleteness of the genome sequence and annotation, our use of poly-A selection, and the use of carrier RNA from another species in one part of the experiment (These are described in more detail in the “Methods”). That said, all factors that had been previously been shown to be maternally localized in *Nasonia* (e.g., *Nv-osk*, *nos*, *dpp*, *cad*, *otd1*, *mad2*, and *gt* [28, 34–36]) were found with high significance in our analyses [33].

### Transcripts with localization in the anterior half of the *Nasonia* early embryos

**Fig. 2:**
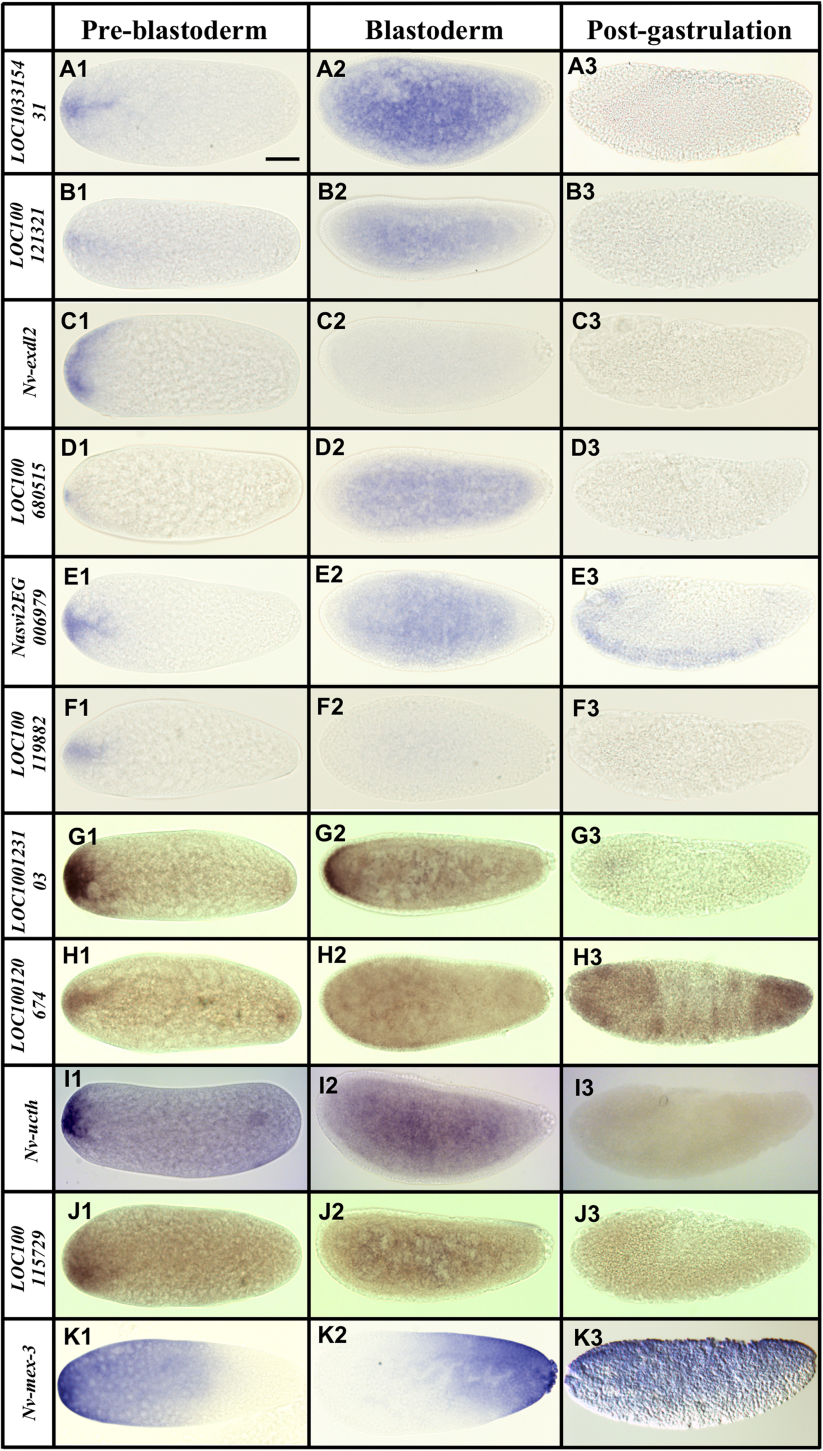
mRNAs localized to the anterior pole at the pre-blastoderm stage. Images of whole mount in situ hybridized embryos for transcripts that showed localization or enrichment at the anterior pole of early (0–2 h) embryos. All embryos are grouped into three columns according to developmental stage (pre-blastoderm, blastoderm, and post-gastrulation). Posterior side is to the right and dorsal side on the top. Scale bar indicates 50 μm

While the anterior factors are not the focus of this manuscript, some interesting observations were made in examining a sample of potentially localized mRNAs. Most transcripts are localized in small domains at the anterior cortex and seem to extend toward the posterior in variable tendrils, rather than being uniform or graded caps of anterior localization (Fig. 2A1–J1, Additional file 1: Figure S1 A, B). A notable exception is the transcript of *Nasonia* homolog of *mex-3*, an RNA-binding protein known for controlling translation of orthologs of the posterior patterning factor *caudal* in the nematode *C. elegans* and the beetle *Tribolium* [37, 38]. *Nv-mex3* mRNA is localized in a broad domain extending far toward the posterior of the embryo (Fig. 2K1), then becomes variable in both the blastoderm and post-gastrular stages (Fig. 2K2, K3).

Transient localization is the most common feature of the anteriorly localized transcripts. Most are ubiquitous or absent by the time the early syncytial blastoderm forms (Fig. 2A2–F2, H2–J3), except for *LOC100313502*, which persists into the blastoderm stage, where it forms an anterior cap (Fig. 2G1, G2).

Among the transcripts showing anterior enrichment, many have predicted functions that may be relevant to egg activation (e.g., homologs of ion channels, including *paralytic*, *slowpoke*, and *piezo* [33]), or anterior-posterior polarity and patterning (e.g., transcription and translation factors such as *mex-3* [33]). There are also a large number of transcripts with no clear homologs outside of *Nasonia* (Fig. 2 and images in Additional file 1: Figure S1) [33]. One of these (*LOC100119982*, Fig. 2F1–F3) is a member of a novel family of ankyrin domain containing molecules that are specific to Chalcid wasps and appear to have obtained a broad diversity of expression and potential function during *Nasonia* development [39].

**Fig. 3.**
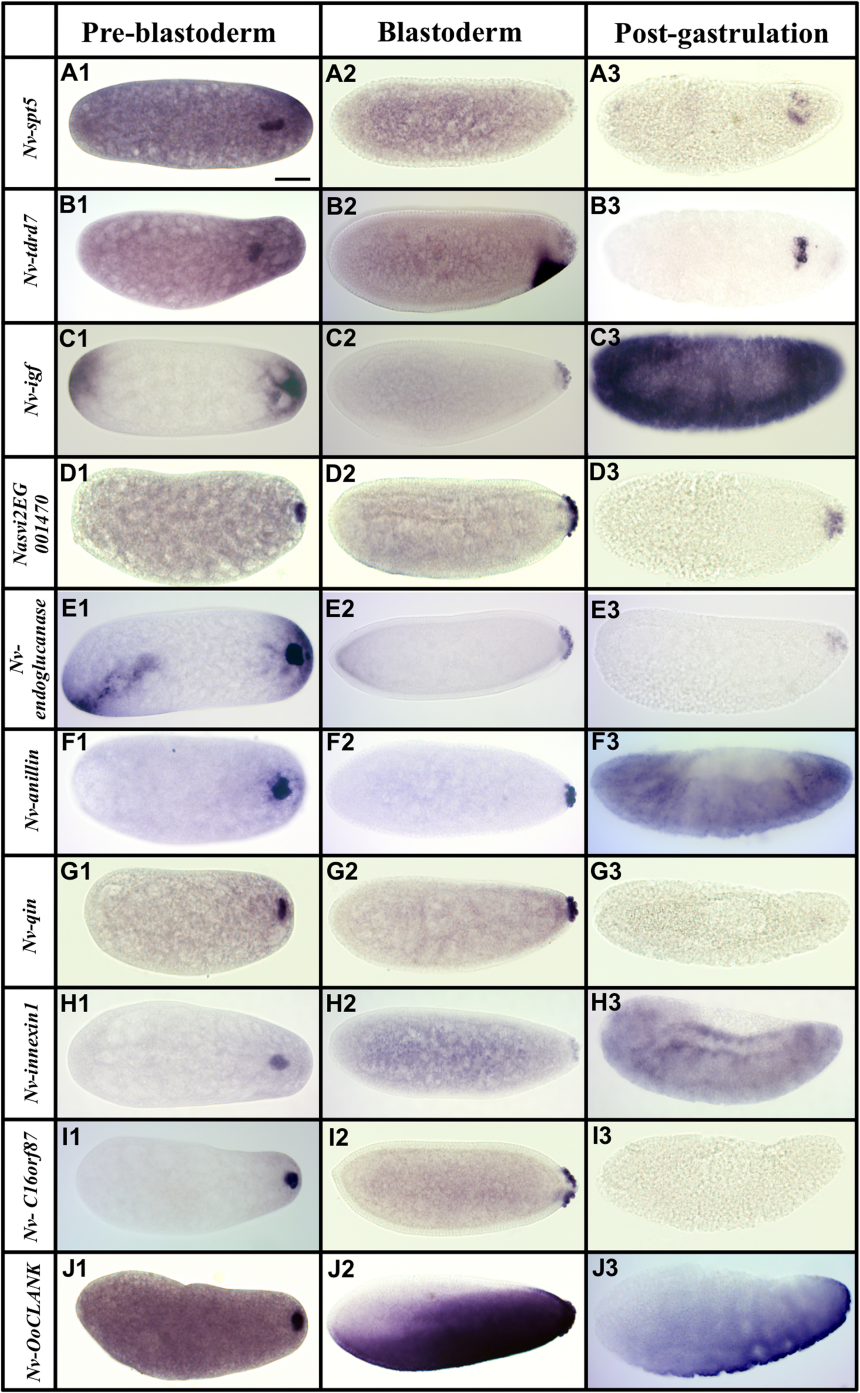
Transcripts localized to the oosome that are subsequently maintained in pole cells**.** Images of whole mount in situ hybridized embryos for transcripts that showed strong enrichment in the oosome in the pre-blastoderm stage, and which subsequently entered, and were maintained in, the pole cells. All embryos are aligned and grouped into three columns (pre-blastoderm, blastoderm, and post-gastrulation) according to their embryogenesis stages, with posterior side to the right and dorsal side on the top. Scale bar indicates 50 μm

Finally, a handful of molecules localized at both poles were detected. These include the previously described *Nv-otd1*, along with *Nv-endoglucanase* (Fig. 3E1–E3), *Nv-insulin-like growth factor* (*Nv-igf*, Fig. 3C1–C3), and *Nv-ucth* (a ubiquitin carboxy terminus hydrolase, Fig. 2I1–I3)). The fact that these were found despite significant concentration of mRNA at both poles that tends to significantly decrease fold differences between the two embryonic halves, further gave confidence that our analysis was robust enough to detect even subtle germ plasm localization of the vast majority of mRNAs.

### Posteriorly localized transcripts in the early *Nasonia* embryo

**Fig. 4:**
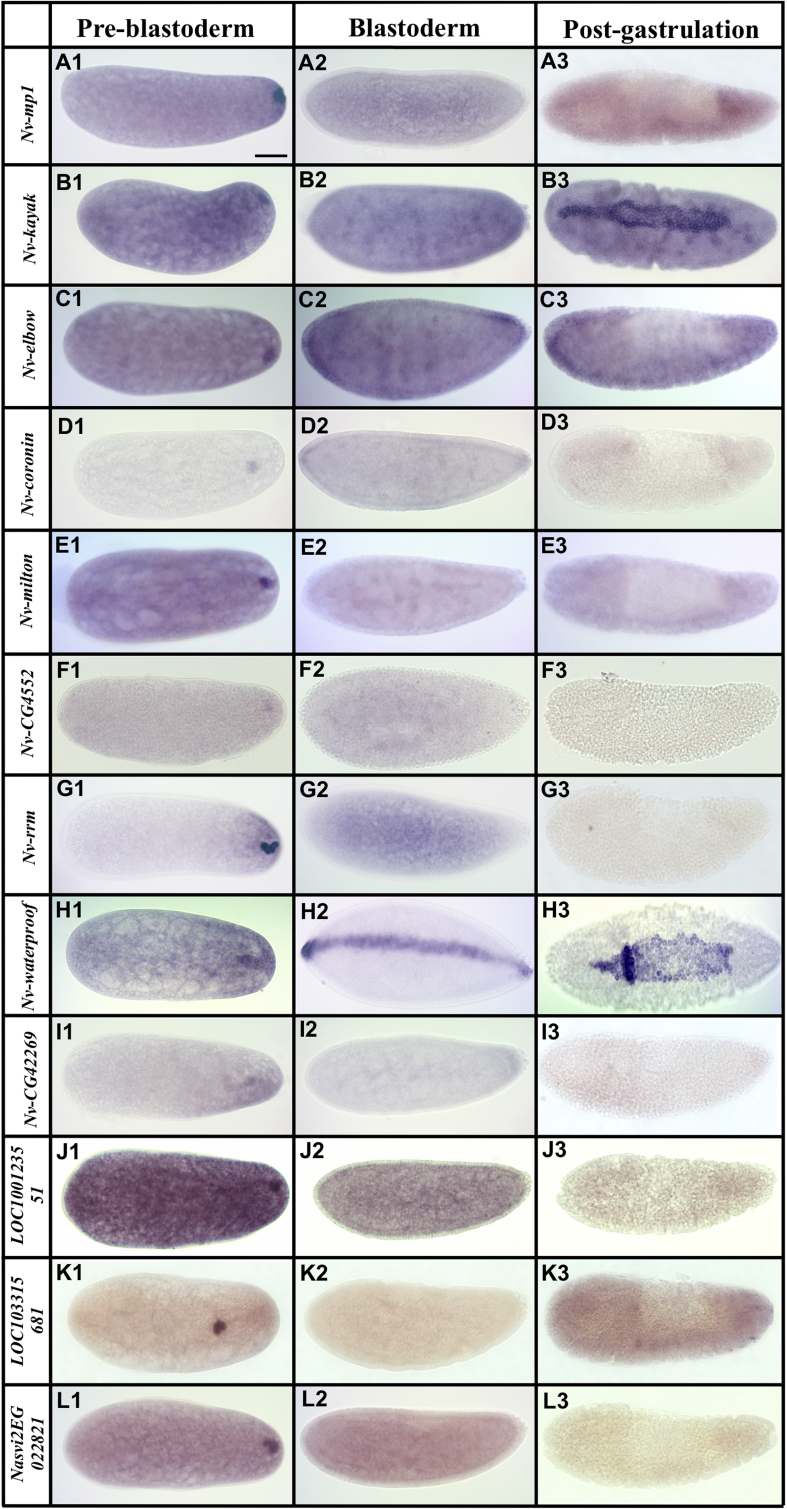
mRNAs localized in the oosome but are not maintained in the pole cells. Images of whole mount in situ hybridized embryos for transcripts that showed strong enrichment in the oosome in the pre-blastoderm stage, but were not maintained in the pole cells. All embryos are aligned and grouped into three columns (pre-blastoderm, blastoderm, and post-gastrulation) according to their embryogenesis stages, with posterior side to the right and dorsal side on the top. Scale bar indicates 50 μm

**Fig. 5:**
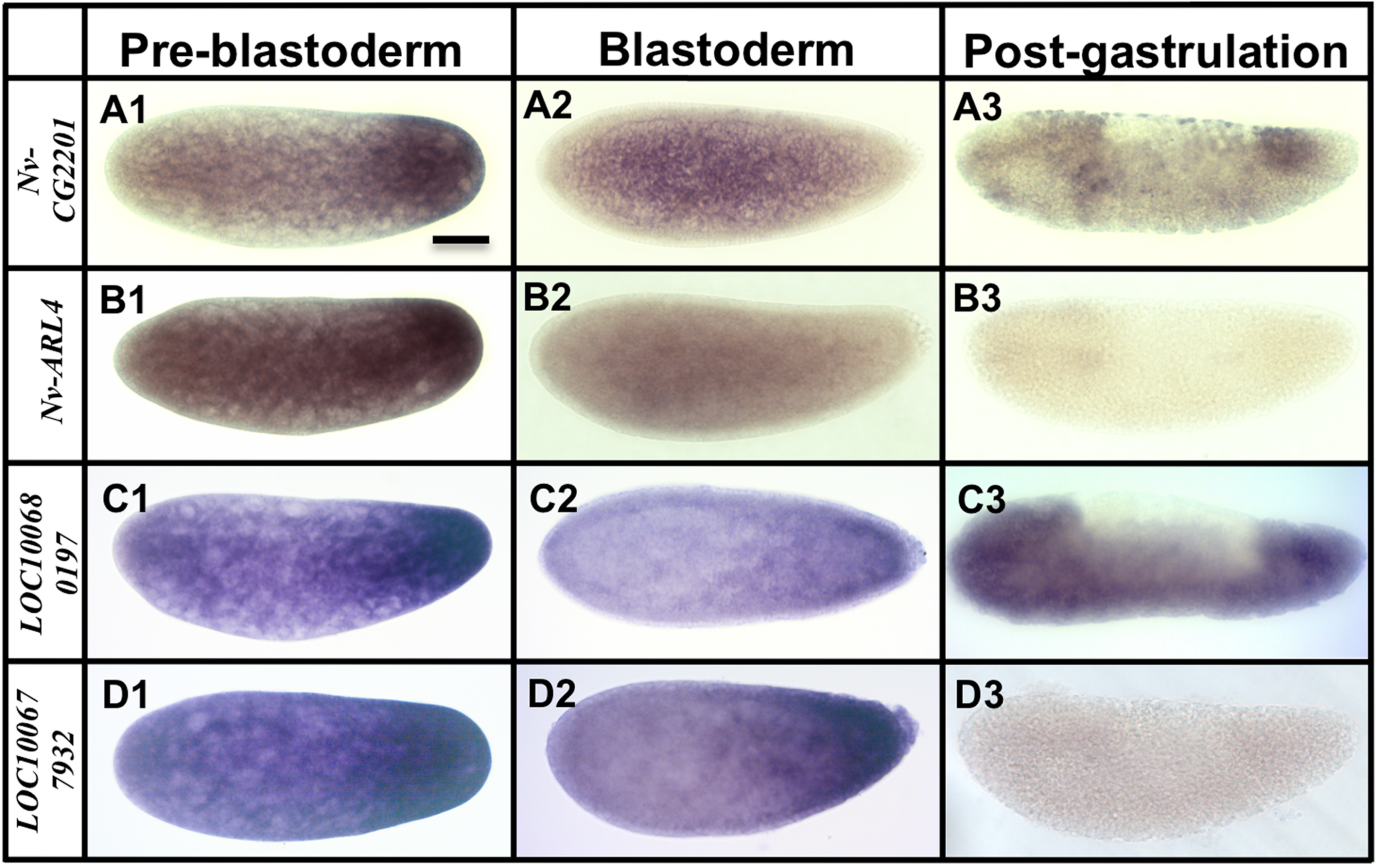
mRNAs strongly enriched in the posterior region of the embryos, but not enriched in the oosome. Images of whole mount in situ hybridized embryos for transcripts that showed some enrichment at the posterior pole, but did not appear to be concentrated in the oosome. All embryos are aligned and grouped into three columns (pre-blastoderm, blastoderm, and post-gastrulation) according to their embryogenesis stages, with posterior side to the right and dorsal side on the top. Scale bar indicates 50 μm

From the two analyses, we identified 90 candidate transcripts that were statistically enriched at the posterior half of the *Nasonia* embryos. We then isolated PCR products for 53 of the most highly enriched posterior genes, and then assessed their localization. We found that 42 of these transcripts are posteriorly localized during pre-blastoderm stage, while the remaining 11 showed no clear enrichment (see Additional file 2: Figure S2). The genes with confirmed posterior enrichment were sorted, based on their temporal and spatial relationship to the oosome and PGCs, into three broad categories: (1) transcripts enriched in the oosome and with their localization maintained in the PGCs (Fig. 3), (2) mRNAs localized in the oosome but not maintained in the PGCs (Fig. 4), and (3) mRNAs strongly enriched in the posterior region of the embryo, but not incorporated in the oosome (Fig. 5, Additional file 1: Figure S1). We also categorized transcripts into broad functional categories, with potential relevance to germ cell function (Additional file 3: Table S1).

#### Transcripts that persist from oosome into pole cells

We identified 11 transcripts that are localized to the oosome and maintained in the PGCs at pole cell formation and beyond. We consider these to be the strongest candidates for having important roles in the specification and function of the PGCs but cannot exclude a priori that they have other (or no) important functions.

Six of the 11 transcripts are maintained in the PGCs beyond the pole cell stage and into the migrating PGCs. They are the *Nasonia* homologs of the fly genes *bark beetle* (*Nv-bark*) (Fig. 1), *spt5* (*Nv-spt5*), *tapas* (*Nv-tdrd7*), *insulin-like growth factor* (*Nv-igf*), and two transcripts without fly homologs (*Nasvi2EG001470* and *Nv-endoglucanase*) (Fig. 3A1–E3).

Among these, *Nv-bark* is the best germline marker, bearing strong and consistent germline association throughout early development, including expression in the late embryonic gonads (Fig. 1). In this respect, it is better than our previously favored marker, *Nv-nos*, which is downregulated significantly toward the end of PGC migration [28]. Bark is a large transmembrane protein, with domains consistent with it being an extracellular receptor and adhesion molecule, with a demonstrated role in stabilizing tricellular junctions in epithelial cells, and is not expressed in the *Drosophila* germline [40, 41]. Since the pole cells are not epithelial in nature, the function of this factor in the oosome is not immediately clear.

*Nv-spt5* is significantly enriched in the oosome and present at low levels in the rest of the embryos in the pre-blastoderm stage (Fig. 3A1). In the blastoderm and migrating germ cell stages, *Nv-spt5* levels are reduced but still enriched in the pole cells while the ubiquitous expression in the embryo persists (Fig. 3A2, A3). Spt5 homologs are involved in regulating RNA polymerase progression during transcription [42], which might suggest that Nv-Spt5 is involved in repressing or otherwise regulating the onset of transcription in the germ cells.

*Nv-tdrd7* is present at appreciable levels throughout the bulk cytoplasm and is also strongly localized in the oosome (Fig. 3B1). This pattern is well reflected in the quantification of mRNA levels in the two halves of the embryo, which show significant numbers of reads coming from the anterior half of the embryo. At the blastoderm stage, *Nv-tdrd7* is moderately enriched in the pole cells and is zygotically expressed in a ventral-posterior patch (Fig. 3B2), which was detected in our earlier analysis of dorsal-ventral patterning [43]. After gastrulation, *Nv-tdrd7* is strongly upregulated in a group of cells that are near to where the germ cells migrate, but it is not clear if they are germ cells, or another cell type present in the same vicinity (Fig. 3B3).

*Nv-igf* is initially enriched at both the anterior and posterior poles (similar to *Nv-otd1* [36]), before becoming specific to the pole cells during the blastoderm stage and the migrating germ cells after gastrulation (Fig. 3C1–C3).

*Nasvi2EG001470* encodes a short peptide of 80 amino acids and was not included in the most recent annotation of the *Nasonia* genome at NCBI, but was present in OGS 2.0 [44]. A very similar sequence is annotated in the close relative *Trichomalopsis* (Accession # OXU26797), suggesting that it is a bona fide transcript that is either novel in the wasps and/or very rapidly evolving. *Nasvi2EG001470* mRNA is strongly enriched in the oosome and pole cells, while levels markedly decrease in migrating germ cells (Fig. 3D1–D3).

Besides its enrichment in the oosome and the pole cells, *Nv-endoglucanase* mRNA is initially localized at both poles during pre-blastoderm stage and early blastoderm stage (Fig. 3E1, E2). Later in blastoderm stage, *Nv-endoglucanase* is downregulated at the anterior pole and becomes specific to the pole cells (Fig. 3E3).

The remaining five of the 11 transcripts that are localized to the oosome and then maintained in the pole cells differ from the preceding set in that they are downregulated after gastrulation (Fig. 3F1–J3). This set includes homologs of *Drosophila anillin* (*scraps*), *qin*, and *innexin1* (*ogre*), (*Nv-anillin*, *Nv-qin*, and *Nv-innexin1*, respectively). A similar temporal pattern was observed for *Nv-osk* [9].

Anillin is an actin-binding protein that localizes to the contractile ring during cytokinesis [45]. In *Drosophila*, Anillin protein is localized in the cleavage furrows when forming the PGCs [46], while its mRNA is ubiquitous. Localization of *Nv-anillin* mRNA to the oosome suggested the hypothesis that Nv-Anillin plays a specialized role in the formation and pinching off of the large, single pole bud in *Nasonia*, which was tested below.

*Nv-qin* encodes a protein containing tudor domains along with an E3 ubiquitin ligase domain. *Qin* is important in processing germline piRNAs, repressing retroelements assembling the nuage, and proper completion of oogenesis in the fly [47–49]. While *qin* has an important late role in germline cells, it is only weakly and diffusely expressed during embryogenesis in *Drosophila* [50]. Its mammalian homolog *RNF17* is required to produce particles in the germline nuage and for sperm development, but not for early germline specification [51]. Given the conserved roles of *Nv-qin*’s orthologs in other species, *Nv-qin* may be required to regulate piRNAs and/or in maintaining the integrity of the oosome as a large particle.

*Nv-innexin1* encodes a putative gap junction protein whose fly homolog is most well-known for its role in proper development and function of the nervous system [52]. Other unexpected roles for Innexin proteins have been described and proposed in insects [53], but at the moment, the potential functional significance of the germline localization in *Nasonia* is unclear.

Two transcripts localized to the oosome and preserved in the pole cells do not have clear *Drosophila* homologs (Fig. 3I1–J3). One of these (*Nv-C16orf87*) encodes a homolog of the human protein *C16orf87* and is enriched in the posterior region and is highly concentrated in the oosome in pre-blastoderm stage (Fig. 3I1–I3). *Nv-C16orf87* encodes a protein belonging to the uncharacterized protein family UPF0547, which contains a zinc-ribbon motif. Functions of this protein and its homologs are unknown.

Finally, another ankyrin domain encoding transcript is strongly localized to the oosome and is taken up into pole cells (Fig. 3J1–J2). It later has a complex and dynamic pattern in the blastoderm stages (Fig. 3J2–J3). This transcript is a member of the newly described CLANK (Chalcid Lineage-specific ANKyrin domain gene) family, of which there are nearly 200 in the *Nasonia* genome [39]. To differentiate from the others, we name it *Nasonia vitripennis Oosome CLANK* (*Nv-OoCLANK*).

#### Transcripts enriched in the oosome but excluded from pole cells

We identified 12 transcripts localized to the oosome but not detected in the pole cells. We define oosome enrichment as staining that is clearly more intense in a spherical region at the posterior pole, while lack of enrichment presents itself as more of uniform cap. We predict that some of these might have roles primarily in the oosome itself, or in the early stages of pole cell formation. It is also likely that some of the transcripts in this set will have roles outside of germline production, such as in embryonic patterning. Such non-germline roles are already known for transiently oosome-localized transcripts of *Nv-dpp*, *Nv-mad2*, and *Nv-cad* [34, 35, 54]. Transiently oosome-associated transcripts whose homologs are well-known developmental factors include *Nv-mp1*, encoding a CLIP protease related to fly *easter* (Fig. 4A1–A3), *Nv-kayak* (Fig. 4B1–B3) encoding a transcription factor downstream of JNK signaling [55], and *Nv-elbow* (Fig. 4C1–C3) encoding a single zinc-finger transcription factor [56].

Several oosome-resident transcripts have suggestive functional annotations. For example, the *Nasonia coronin* gene (*Nv-coronin*, (Fig. 4D1–D3)) encodes a protein whose homologs are known to bind and modulate actin, provide links between the actin and microtubule cytoskeletons, and regulate endo- and exocytosis in several developmental contexts [57, 58]. A germline role for the *Drosophila coronin* ortholog has not been observed.

*Nasonia milton* (*Nv-milton*) is highly enriched in the oosome (Fig. 4E1–E3) and is of particular interest because its *Drosophila* homolog is an adaptor protein that allows mitochondria to be loaded onto, and transported by, microtubule motors [59].

One transient component of the oosome encodes a protein whose fly ortholog is uncharacterized, but whose function may be relevant to oosome function. This is the *Nasonia* homolog of *CG4552* (*Nv-CG4552*), which encodes a protein with a TBC25 domain (Fig. 4F1–F3). Proteins with this domain interact with Rabs to regulate membrane trafficking and dynamics. Such activities have been shown to be crucial for Osk function in the fly [60], and *Nv-CG4552* may play a supporting role in regulating membrane dynamics in the wasp.

Another suggestive localized factor does not have clear orthologs outside of the hymenoptera, but it does have two predicted RNA Recognition Motifs; therefore, we name it *Nv-rrm* (Fig. 4 G1-G3). RRM domains bind RNA and are components of proteins that regulate RNA localization and translation. This novel lineage-specific protein could therefore be involved in the localization of specific RNAs in the oosome or the regulation of translation of specific RNAs within it.

Many of the transiently oosome-localized transcripts do not have annotations that lead to simple hypotheses about potential roles in the germ plasm or PGCs. One of these is *Nv-waterproof* (Fig. 4H1–H3), which encodes a fatty acyl-CoA reductase. *Drosophila waterproof* produces the hydrophobic molecules that coat the tracheal tubes during *Drosophila* embryogenesis and is essential for gas filling of the trachea [61]. Another example is *Nv-CG42269*, which encodes a predicted organic ion transporter protein whose *Drosophila* homolog (*CG42269*) has no described function (Fig. 4I1–I3).

Three oosome-localized transcripts have no clear homologs in *Drosophila* or in other model organisms. *LOC100123551* has a sterile alpha motif (SAM) domain, which might suggest protein-protein or protein-RNA interactions (Fig. 4J1-J3). *LOC103315681* contains a weak similarity to the N-terminal domain of folded gastrulation proteins (but is not a folded gastrulation ortholog) (Fig. 4K1–K3), while *Nasvi2EG022821* has no discernible conserved domains (Fig. 4L1–L3). The functions of these factors will be the object of future investigation. Additional transiently oosome-localized transcripts without predicted functions are shown in Additional file 1: Figure S1 J-Q.

#### Transcripts enriched in the posterior pole but not specifically the oosome

Five transcripts are significantly enriched in the posterior region of the embryo, that do not show significant enrichment within the oosome (Fig. 5). The significance of such transcripts to oosome assembly or to germ cell formation, if any, is not clear. Two transcripts are predicted to encode catalytic enzymes: a choline kinase homologous to the *Drosophila CG2201* (*Nv-CG2201*), and a homolog of the ADP ribosylation factor-like 4 protein (*Nv-ARL4*). Finally, *LOC100680197* and *LOC100677932* have no identifiable homologs outside of hymenoptera. *LOC100680197* encodes a protein with MYND-type zinc-fingers and a p27-like domain, while *LOC100677932* has no clear conserved or functional domains. Additional images of posteriorly enriched transcripts are shown in Additional file 1: Figure S1 R-X.

### Functional analysis of a sample of oosome-localized transcripts

While localization of an mRNA to the oosome and pole cells may suggest a function related to PGC specification or the specialized cell biological processes of pole cell formation, demonstration of any such function would be required to demonstrate the functional relevance. We chose a sampling of five promising molecules for in-depth functional analysis (*Nv-bark*, *Nv-anillin*, *Nv-rrm*, *Nv-coronin*, and *Nv-innexin1*). These genes were chosen because we predicted they could produce phenotypes that would be easily scorable and interpretable when knocked down. For all of these except *Nv-rrm*, we believed that the predicted functions of the proteins as components or modulators of the cytoskeleton suggested that they would be important for the movement of the oosome in the early embryo and/or the formation of the pole cells. *Nv-rrm* was chosen because of its novelty and the possibility that its predicted RNA-binding function would be important in maintaining the structure of the oosome.

#### Parental RNA interference

We initially tried to apply our parental RNAi (pRNAi) approach [36], but quickly found that this was not the ideal approach. Most dsRNAs caused reduced fecundity, with most of the obtainable eggs being apparently normal escapers. Eventually, we found injection conditions which produced embryos with phenotypes in a very low penetrance (2–6%) from three of the five genes (*Nv-rrm*, *Nv-coronin*, and *Nv-innexin1)* chosen for functional analysis. Embryos showing phenotypes had strongly reduced or absent pole cells, with disorganized germ plasm residue at the posterior pole of the embryos in the blastoderm stage (see Additional file 4: Figure S3). Infrequently, *Nv-coronin* knockdown embryos were characterized by pole cells that did not migrate to the gonad, but instead remained at the pole after gastrulation (Additional file 4: Figure S3 D, F).

We were not able to achieve even these modest results with pRNAi for *Nv-bark* and *Nv-anillin*. At dsRNA concentrations from 1.5 to 2.5 μg/μL, injection led to no eggs being laid. At lower concentrations (250 ng/μL, 500 ng/μL, and 750 ng/μL), all laid eggs developed normally. Since embryos showing phenotypes were either completely absent or extremely rare for our genes of interest, it became necessary to develop a new technique to assess the functions of the novel oosome genes we discovered.

#### Embryonic injection of dsRNA

To circumvent the low penetrance problem from the pRNAi, we developed a protocol for embryonic injection of dsRNA (eRNAi) followed by fixation and in situ hybridization (see details in “Methods”). By using this newly developed protocol, we were able to achieve much higher penetrance with most embryos showing phenotypes consistent with knockdown of the target gene (see description below).

**Fig. 6:**
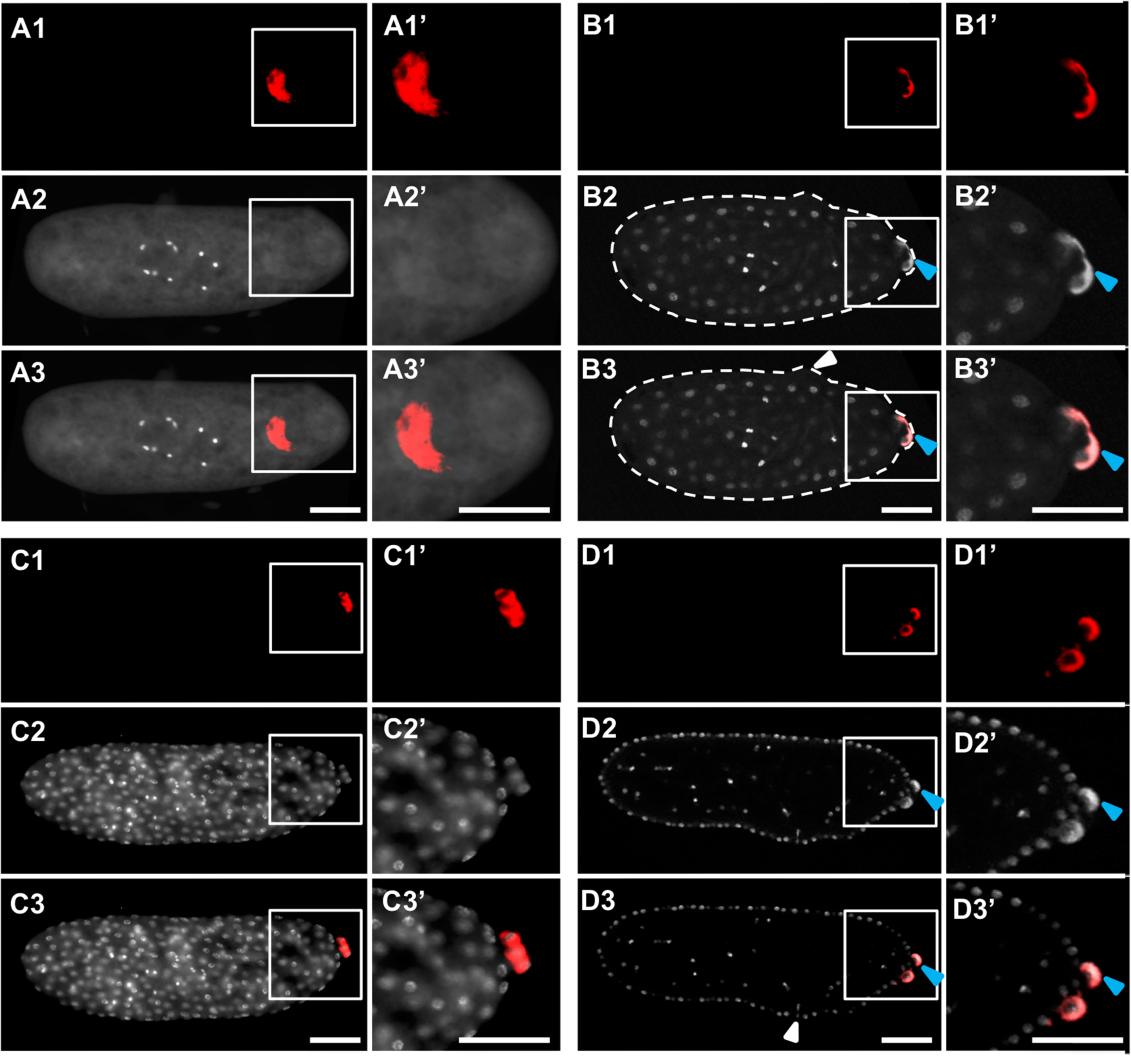
Development of embryos injected with eGFP dsRNA and probed with *Nv-bark*. **A1–A3’** Pre-blastoderm stage. **B1–B3’** Early blastoderm stage. **C1**–**C3’** Mid-blastoderm stage. **D1–D3’** Mid-blastoderm stage. In each quadrant, the topmost image (**A1**, **B1**, **C1**, **D1**) shows a single channel image of fluorescent in situ hybridization signal using a probe against *Nv-bark beetle*. The next image down contains single-channel images of DAPI staining (**A2**, **B2**, **C2**, **D2**). The merges of two single channels are in the bottom image of each quadrant (**A3**, **B3**, **C3**, **D3**). Enlarged views of the posterior pole (marked by white boxes) appear to the right of each image (**A1’**–**D3’**). The white dashed lines in **B2** and **B3** mark the edges of the embryos. The white arrowheads indicate areas of damage in the embryos. Some staining produced appreciable bleed-through from the fluorescent in situ detection into the DAPI channel, and instances of this are marked with blue arrow heads. All embryos are positioned with posterior side to the right and dorsal side on the top. Scale bar indicates 50 μm

**Fig. 7.**
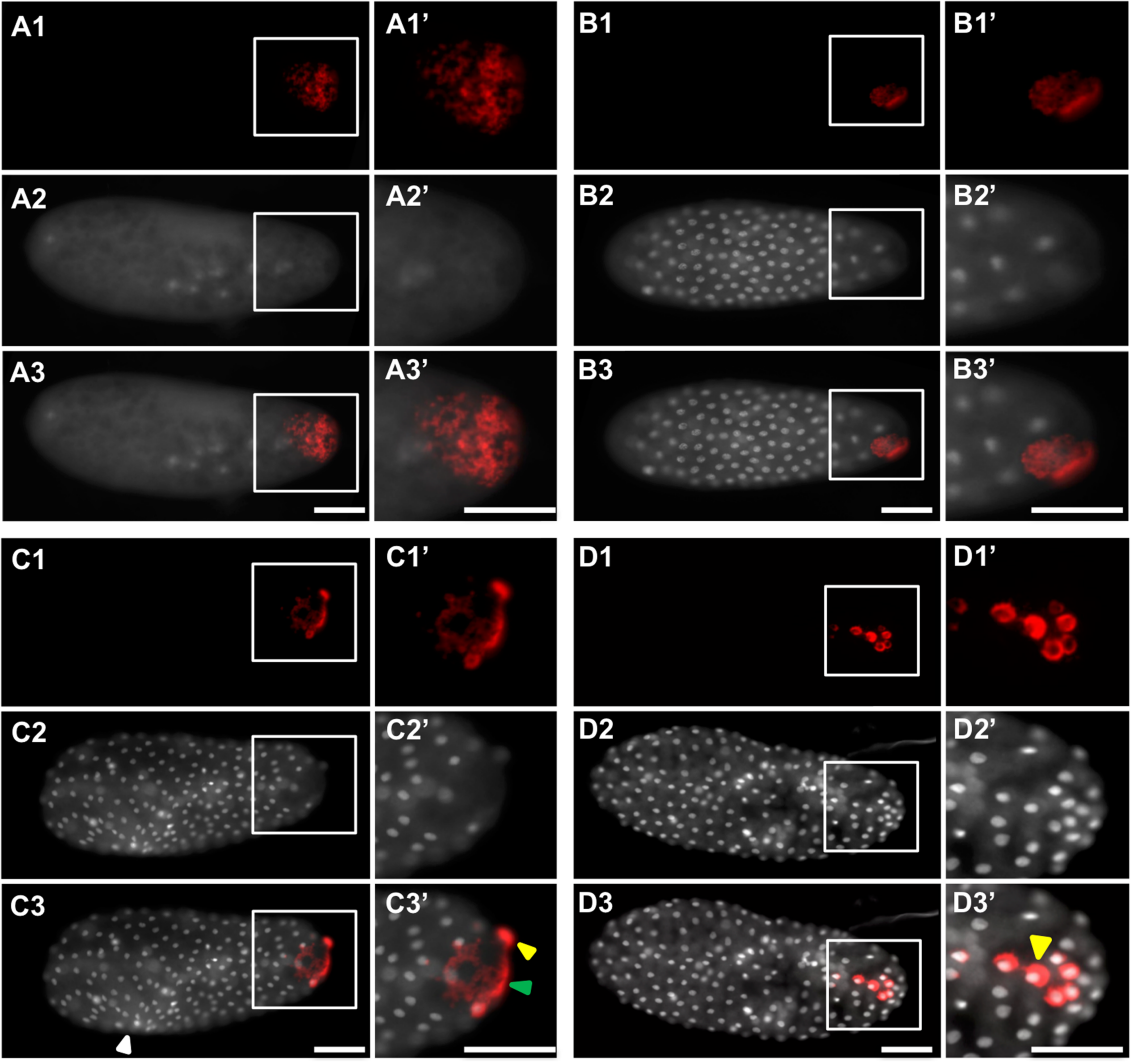
eRNAi phenotypes in *Nv-bark beetle* dsRNA-injected embryos. **A1**–**A3’** Pre-blastoderm stage. **B1**–**B3’** Early blastoderm stage. **C1**–**C3’** Mid-blastoderm stage. **D1**–**D3’** Mid-blastoderm stage. In each quadrant, the topmost image (**A1**, **B1**, **C1**, **D1**) shows a single-channel image of fluorescent in situ hybridization signal using a probe against *Nv-nanos*. The next image down contains single-channel images of DAPI staining (**A2**, **B2**, **C2**, **D2**). The merges of two single channels are in the bottom image of each quadrant (**A3**, **B3**, **C3**, **D3**). Enlarged views of the posterior pole (marked by white boxes in the whole embryo panels) appear to the right of each image (**A1’**–**D3’**). The white arrowheads indicate areas of injection damage in the embryos. The green arrow head indicates the free germ plasm in the embryo, and yellow arrowheads indicate germ plasm associated with a nucleus. All embryos are positioned with the posterior side to the right and dorsal side on the top. Scale bar indicates 50 μm

**Fig. 8.**
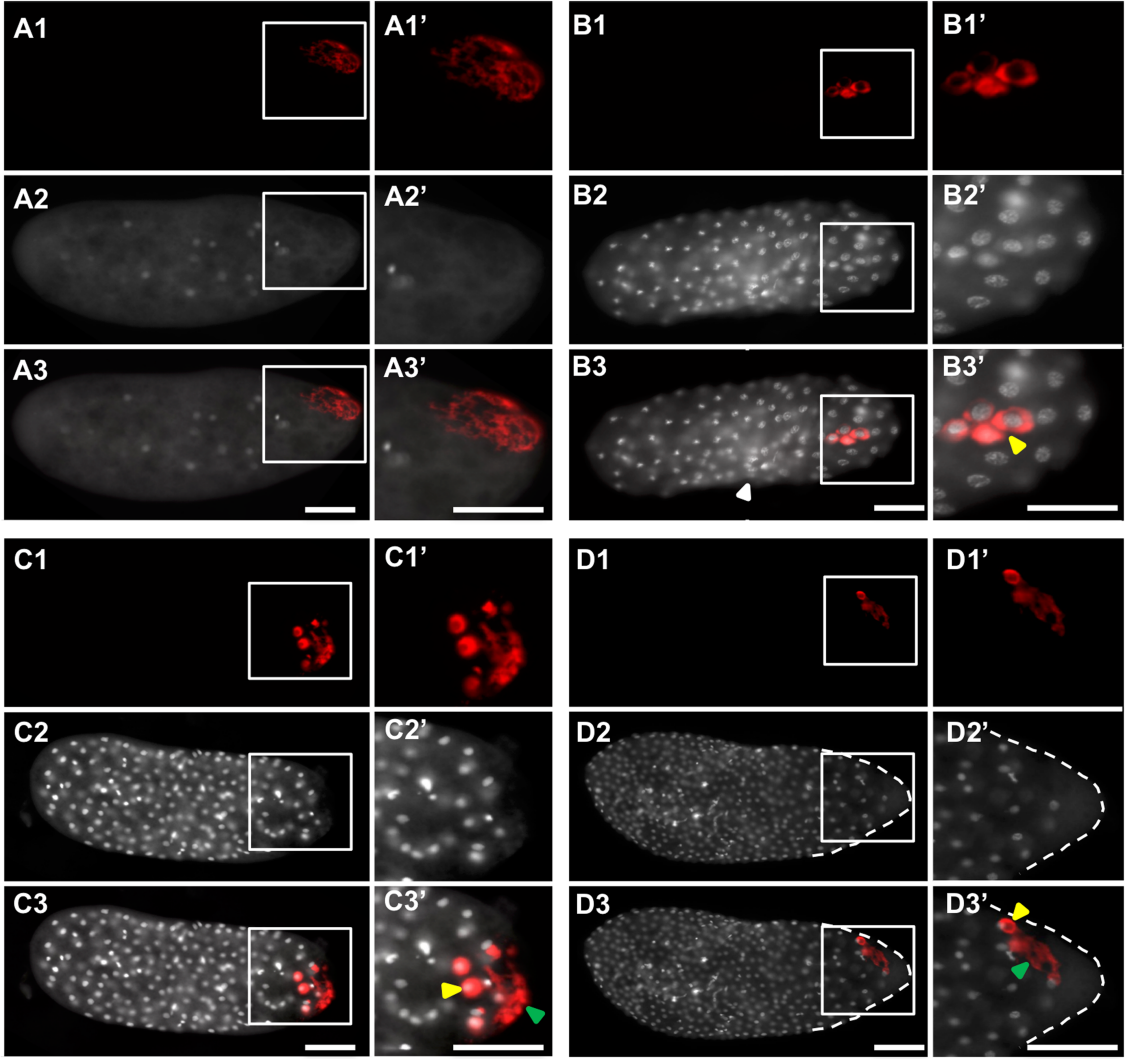
eRNAi phenotypes in *Nv-anillin* dsRNA-injected embryos. **A1**–**A3’** Pre-blastoderm stage. **B1**–**B3’** Mid-blastoderm stage. **C1**–**C3’** Mid-blastoderm stage. **D1**–**D3’** Mid-blastoderm stage. In each quadrant, the topmost image (**A1**, **B1**, **C1**, **D1**) shows a single-channel image of fluorescent in situ hybridization signal using a probe against *Nv-bark beetle*. The next image down contains single-channel images of DAPI staining (**A2**, **B2**, **C2**, **D2**). The merges of two single channels are in the bottom image of each quadrant (**A3**, **B3**, **C3**, **D3**). Enlarged views of the posterior pole (marked by white boxes in the whole embryo panels) appear to the right of each image (**A1’**–**D3’**). The yellow arrowheads indicate that the germ plasm associated with a nucleus. The green arrow heads indicate the free germ plasm in the embryos. The white arrowheads indicate areas of injection damage in the embryos. All embryos are positioned with the posterior side to the right and dorsal side on the top. Scale bar indicates 50 μm

**Fig. 9.**
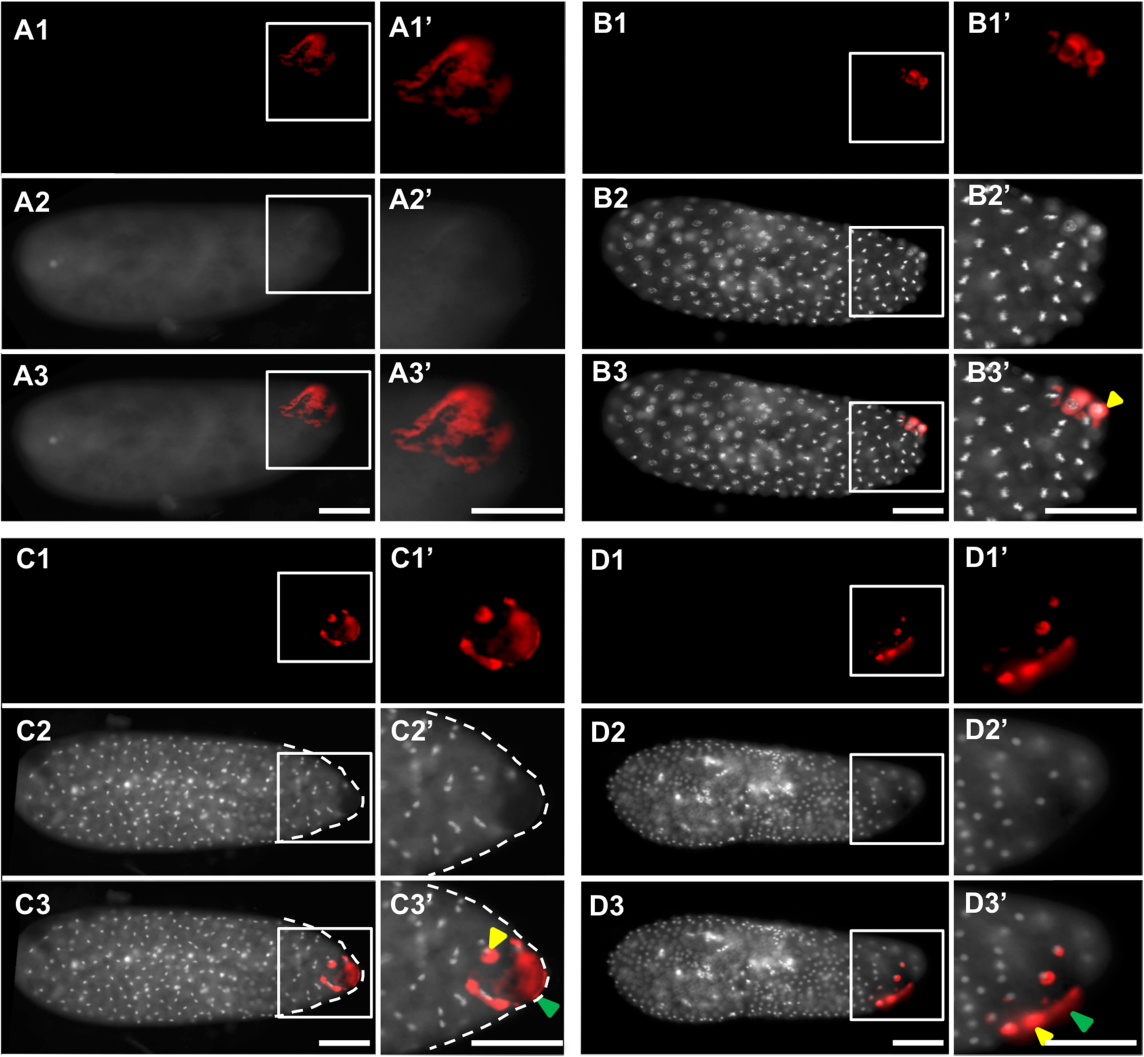
eRNAi phenotypes in *Nv-rrm* dsRNA-injected embryos. **A1**–**A3’** Pre-blastoderm stage. **B1**–**B3’** Mid-blastoderm stage. **C1**–**C3’** Mid-blastoderm stage. **D1**–**D3’** Mid-blastoderm stage. In each quadrant, the topmost image (**A1**, **B1**, **C1**, **D1**) shows a single-channel image of fluorescent in situ hybridization signal using a probe against *Nv-bark beetle*. The next image down contains single-channel images of DAPI staining (**A2**, **B2**, **C2**, **D2**). The merges of two single channels are in the bottom image of each quadrant (**A3**, **B3**, **C3**, **D3**). Enlarged views of the posterior pole (marked by white boxes in the whole embryo panels) appear to the right of each image (**A1’**–**D3’**). The yellow arrowheads indicate that the germ plasm associated with a nucleus. The green arrow heads indicate the free germ plasm in the embryos. The white arrowheads indicate areas of injection damage in the embryos. All embryos are positioned with the posterior side to the right and dorsal side on the top. Scale bar indicates 50 μm

**Fig. 10.**
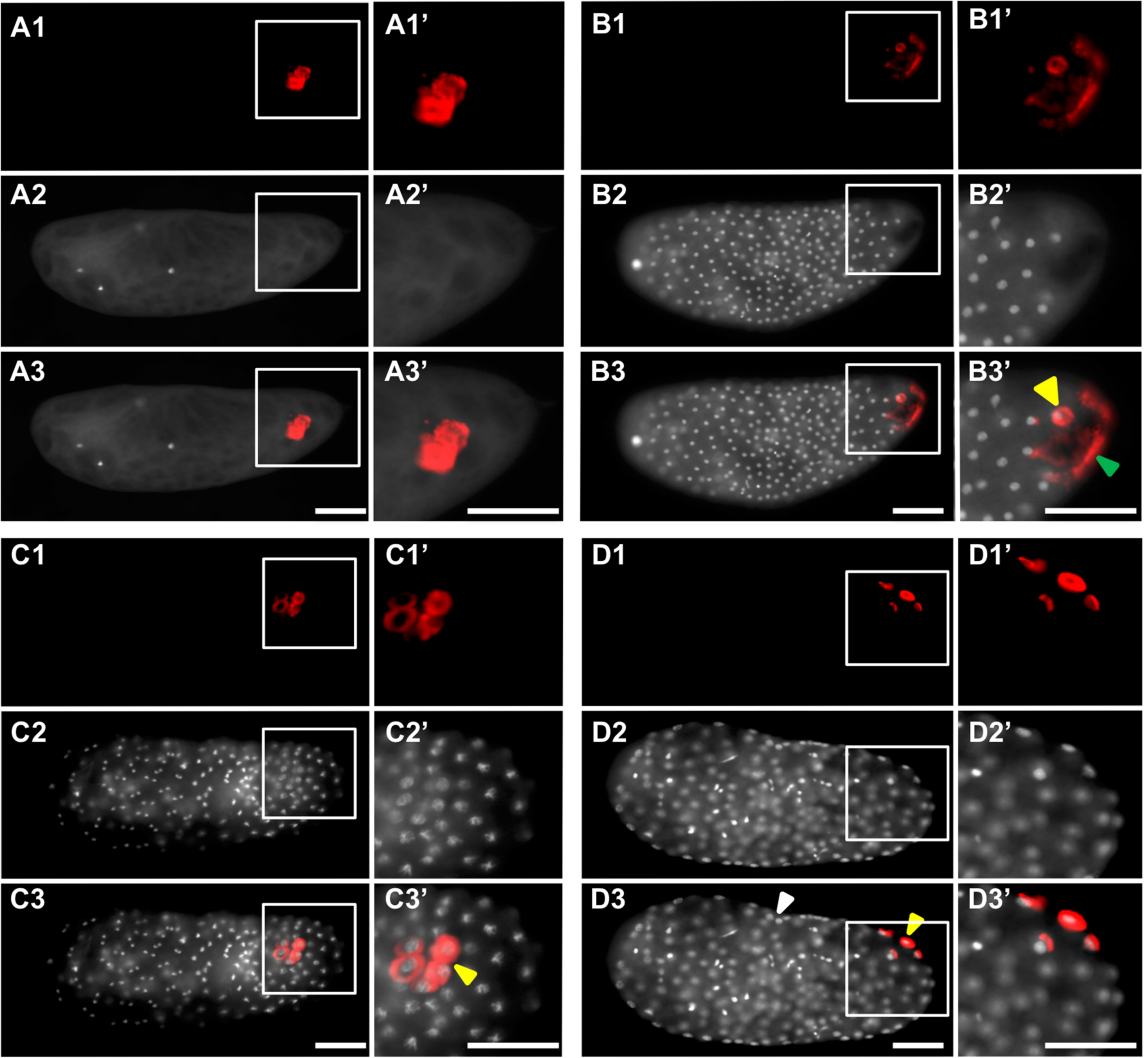
eRNAi phenotypes in *Nv-coronin* dsRNA-injected embryos. **A1**–**A3’** Pre-blastoderm stage. **B1**–**B3’** Mid-blastoderm stage. **C1**–**C3’** Mid-blastoderm stage. **D1**–**D3’** Mid-blastoderm stage. In each quadrant, the topmost image (**A1**, **B1**, **C1**, **D1**) shows a single-channel image of fluorescent in situ hybridization signal using a probe against *Nv-bark beetle*. The next image down contains single-channel images of DAPI staining (**A2**, **B2**, **C2**, **D2**). The merges of two single channels are in the bottom image of each quadrant (**A3**, **B3**, **C3**, **D3**). Enlarged views of the posterior pole (marked by white boxes in the whole embryo panels) appear to the right of each image (**A1’**–**D3’**). The yellow arrowheads indicate that the germ plasm associated with a nucleus. The green arrow heads indicate the free germ plasm in the embryos. The white arrowheads indicate areas of injection damage in the embryos. All embryos are positioned with posterior side to the right and dorsal side on the top. Scale bar indicates 50 μm

However, the physical injection and harsher fixation methods required by this technique can lead to disrupted embryonic morphology. We identified two major types of damage: (1) a rougher blastoderm surface (for example Fig. 6B3, Fig. 7D3) and less evenly spaced syncytial nuclei (for example, Fig. 6D3, Fig. 8C3, Fig. 9C3, Fig. 10C3). Since these morphological defects were found in both control and knockdown embryos, we treated them as artifacts and did not consider them as potential gene-specific phenotypes. Reciprocally, any phenotypes that were observed consistently in the knockdown of a gene of interest, but were never observed in the negative control, were considered to be caused by the reduction of the gene of interest. We confirmed that target levels were reduced by eRNAi using qPCR (Additional file 5: Figure S4).

We cannot exclude that the genes knocked down here have broader functions outside of the oosome and pole cells that are obscured by the embryo-wide artifacts. However, since our goal with these knockdowns was to simply show the functional relevance of a sample of the genes localized to the oosome, such functions are peripheral to this manuscript.

Crucially, despite the non-specific artifacts described above, embryogenesis, including germ cell specification, was not significantly disrupted in negative control eGFP eRNAi. Specifically, the oosome in pre-blastoderm stage was as intact as in the wild-types (Fig. 6A1–A3’, compared to Fig. 1D), the oosome flattened a thin layer and filled in the bud at the beginning of the blastoderm stage (Fig. 6B3, compared to Fig. 1E), and the pole cells were successfully formed in the early blastoderm stage (Fig. 6C1–D3’, compared to Fig. 1F).

#### eRNAi reveals three genes involved in maintaining integrity of the oosome

*Nv-bark* is the transcript most strongly and consistently associated with PGC specification over embryonic development that we have found (Fig. 1). However, its potential function in the germline is not clear. Since it encodes a transmembrane protein involved in epithelial tricellular junctions in *Drosophila* [40], we speculated that it might have a role in mediating adhesion or migration of the pole cells once they were formed. In contrast to this expectation, we observed very early defects in embryos where this gene was knocked down. In pre-blastoderm syncytial embryos (before migration of nuclei to periphery, corresponding to wild-type stages shown in Fig. 1C, D), the oosome (detected by *Nv-nos* in situ hybridization in this case) loses its integrity as a dense and spherical unit and appears more as a disorganized film at variable locations along the embryonic cortex near the posterior pole (Fig. 7A1–A3’, compared to Fig. 1B–D and Fig. 6A1–A3’). This cortical localization is distinct from the normal position of the oosome in the central column of the cytoplasm (note that in wild-type and control the migrating oosome is in the same focal plane as the pre-blastoderm nuclei).

This film of disrupted oosome material remains attached to the cortex throughout the time where nuclei reach the cortex and pole cells would have normally formed (Fig. 7B1–B3’). When nuclei fully penetrate the cortex, the germ plasm surrounds nuclei in a way similar to what is seen in pole cells (Fig. 7C1–C3’, D1–D3’). However, these nuclei remain part of the single-layer embryonic syncytium, do not form clusters, and are not isolated from the rest of the syncytium, in contrast to normal pole cells (Fig. 7C1–D3’, compared to Fig. 1F-G, and Fig. 6C1–D3’). In addition, the association of nuclei with disrupted oosome material often does not occur directly at the pole, but rather in lateral positions. We have never observed pole cells forming away from the pole in either wild-type or negative control embryos (Fig. 7D1–D3’, compared to Fig. 1F-G, and Fig. 6C1–D3’).

Another oosome-localized transcript that caught our attention early in our analysis was the homolog of *anillin* (*Nv-anillin)*. mRNA of *Drosophila anillin* is not localized to the polar granules [62], but is present evenly throughout the embryo ( [62, 63]). Anillin protein, however, accumulates at the base of pole cells when they are budding in *Drosophila* [46]. Since Anillin is a major component of the contractile ring during mitotic cytokinesis [45] and is enriched at the bud furrow during pole cell formation in *Drosophila* [46], we predicted that the enrichment of *Nv-anillin* mRNA in the oosome and pole cells would be related to *Nasonia*’s unique way of forming a single large pole bud instead of several small ones, as occurs in *Drosophila*.

Unexpectedly, the phenotype of *Nv-anillin* is very similar to that of *Nv-bark*. The oosome loses it integrity (Fig. 8A1–A3’, compare to Fig. 7A1–A3’) and pole cells are not formed. Instead, disrupted oosome material (here marked by *Nv-bark* in situ hybridization) remains bound to the posterior-lateral cortex of the embryo, where it interacts with nuclei penetrating the cortex (Fig. 8B1–C3’, compared to Fig. 7B1–D3’). Interestingly, partial posterior budding is occasionally observed in *Nv-anilli*n knockdown embryos and appears to persist longer (note larger number of nuclei in Fig. 8D1–D3’ compared to Fig. 6C1–D3’, and Fig. 1G) without completing budding. In addition, the disrupted oosome material does not enter the bud, but instead it remains attached to the posterior-lateral cortex (Fig. 8D1–D3’).

A similar set of phenotypes was observed when we knocked down *Nv-rrm* with eRNAi. The oosome lacked integrity and was attached to the embryonic cortex in pre-blastoderm embryos (Fig. 9A1–A3’), and pole cells did not form normally (Fig. 9B1–B3’), resulting in excess disrupted oosome material at the cortex (Fig. 9C1–C3’, D1–D3’). In addition, we observed late/prolonged posterior protrusion in the blastoderm stage (Fig. 9C1–C3’), similar to what was seen for *Nv-anillin* knockdown (Fig. 8D1–D3’). In contrast to control and wild-type, the bud did not take up the oosome and did not pinch off to form pole cells. Unusually for the results of eRNAi presented here, the nuclei associated with oosome material in the embryo shown in Fig. 9B1–B3’ are in a different phase of the cell cycle from the surrounding nuclei, which would be a typical property of pole cells. However, they are not tightly clustered, are isolated from the rest of the blastoderm, and do not form a separate layer on the surface of the blastoderm. This phenotype may represent a less complete knockdown of *Nv-rrm* than observed in other embryos.

It is important to note at this point that the common phenotype of the above three knockdowns is not the same as the complete loss of germ plasm activity. Such phenotypes are seen for *Nv-osk* and *Nv-vas.* When these genes are knocked down, posterior mRNAs such as *Nv-nos* take on a uniformly graded posterior cap and no enriched accumulation of germ plasm markers is ever observed at the posterior [9, 28]. This suggests that *Nv-rrm*, *Nv-bark*, and *Nv-annilin* are involved in assembling the oosome into a single large particle and/or maintaining this morphology throughout early embryogenesis. However, they are not essential for all properties of the *Nasonia* germ plasm. Determining the detailed molecular basis of these phenotypes and how the three genes examined above interact in the oosome and pole cells will be a long-term project.

Finally, large posterior protrusions, similar to the one that initiates pole cell formation in wild-type (Fig. 1E) and control (Fig. 6B3, C3), were observed after eRNAi against *Nv-annilin* and *Nv-rrm* (Fig. 8D1–D3’, Fig. 9C1–C3’). This is in spite of the fact that the oosome does not form and does not move normally in the central column of the embryo in the knockdowns. The dissociation of the posterior protrusive budding and the formation of the PGCs suggests that neither the oosome, nor its remnants, induce the initial protrusion that leads to budding. This has some precedent in *Drosophila*, where the autonomous ability of the fly embryo to produce pole cell-like structures at both poles of the embryo is revealed when Arf guanine exchange factor Steppke is reduced [64]. However, in the fly, normal global repression of pole cell formation is overcome by germ plasm components (primarily *germ-cell-less*), while in *Nasonia*, at least the initial budding appears to be germ plasm independent.

In conclusion, these three gene knockdowns produce very similar phenotypes to one another, suggesting that they are all required for oosome integrity and inducing proper pole cell formation. Understanding the exact molecular mechanisms and interactions underlying this is beyond the scope of this manuscript, but will be an area of intense research in the future.

#### RNAi against Nv-coronin and Nv-innexin1 does not affect the oosome, but disrupts pole cell formation

**Fig. 11.**
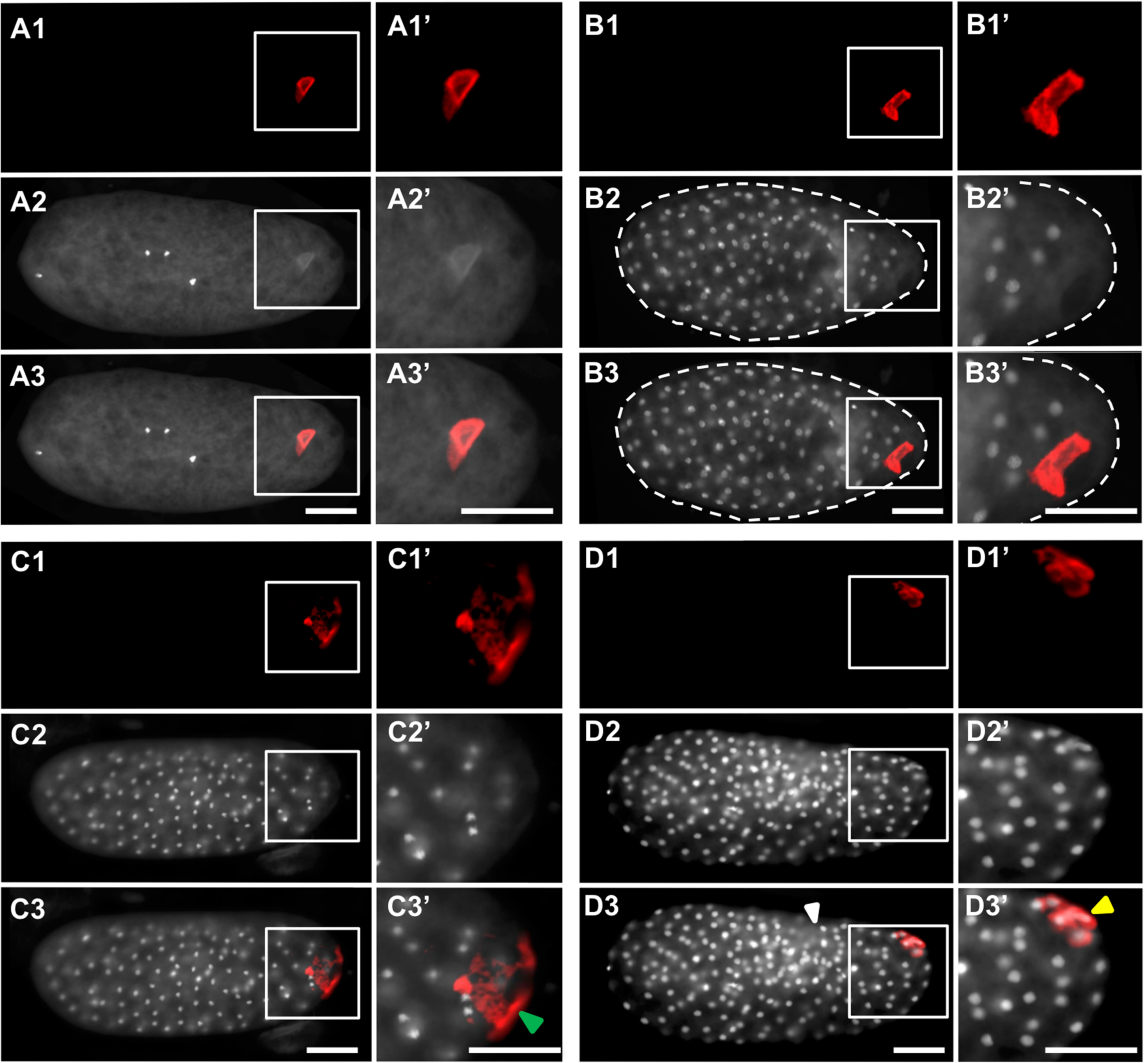
eRNAi phenotypes in *Nv-innexin1* dsRNA-injected embryos. **A1**–**A3’** Pre-blastoderm stage. **B1**–**B3’** Mid-blastoderm stage. **C1**–**C3’** Mid-blastoderm stage. **D1**–**D3’** Mid-blastoderm stage. In each quadrant, the topmost image (A1, B1, C1, D1) shows a single-channel image of fluorescent in situ hybridization signal using a probe against *Nv-bark beetle*. The next image down contains single-channel images of DAPI staining (**A2**, **B2**, **C2**, **D2**). The merges of two single channels are in the bottom image of each quadrant (**A3**, **B3**, **C3**, **D3**). Enlarged views of the posterior pole (marked by white boxes in the whole embryo panels) appear to the right of each image (**A1’**–**D3’**). The yellow arrowheads indicate that the germ plasm associated with a nucleus. The green arrow heads indicate the free germ plasm in the embryos. The white arrowheads indicate areas of injection damage in the embryos. All embryos are positioned with the posterior side to the right and dorsal side on the top. Scale bar indicates 50 μm

We tested the functions of two other transcripts with eRNAi: *Nv-coronin* and *Nv-innexin1*. Knockdown of both genes left the oosome intact and able to migrate through the embryonic cytoplasm normally (Fig. 10A1–A3’, Fig. 11A1–A3’). However, in both cases, pole cell budding fails, leading to phenotypes in the blastoderm that are very similar to those in the knockdowns of *Nv-bark*, *Nv-anillin*, and *Nv-rrm*: disrupted oosome material remains in the embryos and pole cell formation fails (Fig. 10B1–D3’, Fig. 11B1–D3’). The similarity of the phenotypes at the blastoderm stage suggests that the partial uptake of oosome material, and misplacement of germ plasm-interacting nuclei is mostly a downstream consequence of the failure to form pole cells, which appears to be an indirect result of *Nv-bark*, *Nv-anillin*, and *Nv-rrm* knockdown, and a more direct result of *Nv-coronin* and *Nv-innexin* knockdown. Understanding the cellular basis of *Nv-coronin* and *Nv-innexin* knockdown phenotypes will require in-depth analysis that is again beyond the scope of this manuscript.

## Discussion

### RNAseq analyses

Our results have uncovered an unexpectedly large divergence in the mRNA content of the germline determinant of the wasp *Nasonia vitripennis* relative to the composition of the polar granules in *Drosophila melanogaster*. This was achieved using RNAseq followed by statistical detection of differential enrichment of mRNAs between the anterior and posterior poles of the early embryo. The statistical predictions were then tested by in situ hybridization, and we validated the functional relevance of a subset of the localized transcripts using RNAi. Overall, these results provided insights into the essential properties of insect germ plasm and how different molecules can be deployed to carry out conserved functions. In addition, our functional results provide a basis for a future detailed understanding of how the unusual form of the wasp oosome is generated and maintained.

### Discovery of potential functional analogs to crucial *Drosophila* germ components in the *Nasonia* oosome

Our analyses found that *Nv-nos*, *Nv-osk* (already known factors found in both oosome and polar granules), and *Nv-ovo* were the only mRNA factors with conserved germ plasm localization between fly and wasp (our data compared to [62, 65]). We also found *Nv-aub* whose fly ortholog is localized as protein, but not mRNA, in *Drosophila* [66]. Thus, there are at least two crucial localized *Drosophila* germ plasm transcripts whose homologs are not maternally provided as mRNA in *Nasonia*, and dozens of *Nasonia* transcripts that do not have obvious homologs or functional analogs.

A prime example is a *polar granule component* (*pgc*), which encodes a small peptide [67], and whose mRNA is strongly localized to the posterior pole [68]. Polar granule component protein has a crucial role in the global repression of transcription that occurs in pole cells upon their formation, through an interaction with the transcription elongation factor TEF-b [67]. This repression is a widely conserved feature of PGCs across animals, which makes it somewhat surprising that *pgc* appears to be a novelty in the *Drosophila* lineage [68].

One explanation may be that TEF-b orthologs have been shown to be crucial for transcriptional quiescence in disparate model systems, but with diverse interaction partners. The TEF-b ortholog in the worm *C. elegans* interacts with the novel germline factor PIE-1 to repress transcription in the nematode germline [69]. In humans, TEF-b phosphorylates the basal transcription factor Spt5, converting it from transcriptional inhibitor into an activator [70]. Interestingly, mRNA for the *Nasonia* Spt5 homolog (*Nv-spt5*) (Fig. 3A1–A3) is localized strongly to the oosome and pole cells. If an interaction between Nv*-*Spt5 and the Nasonia TEF-b homolog has a role in regulating the cessation of transcription in *Nasonia* pole cells, it would be strong evidence for TEF-b being a core, conserved component of the germline fate, whose interaction partners and regulators are labile across lineages.

Another crucial *Drosophila* germ cell factor that is not present in the *Nasonia* oosome is *germ cell less* (*gcl*). The Gcl protein itself is very highly conserved at the sequence level in *Nasonia*, but the mRNA showed no enrichment in our RNAseq experiments [33]. Gcl is important for the proper production of *Drosophila* pole cells, apparently by regulating the orientation centrosome separation at the posterior pole, which is required for efficient pole cell formation and uptake polar granules by the pole cells [71]. At the molecular level, Gcl seems to act by downregulating Torso signaling, to allow the proper conditions for pole cells to form [72]. The lack of Gcl function in the germline of *Nasonia* is consistent with the lack of Torso signaling at the termini in the wasp [73], making the need for Gcl in this process redundant. At the moment, it is not clear whether the use of Gcl in pole cell formation is a recent novelty in *Drosophila*, or whether it was present ancestrally, but lost in the Hymenopteran lineage, where lack of terminal Torso signaling is a widespread feature [74].

### Oosome-localized factors with potential roles in conserved features of germ cells

A general feature of germ cells and germ plasm is an enriched endowment of mitochondria [75, 76]. In *D. melanogaster*, the “long Osk” isoform plays an important role in concentrating mitochondria in the pole plasm [77]. But, since long Osk appears to be a novelty of *D. melanogaster* and its close relatives [9], other molecules should be expected to perform this role in other species. Suggestively, mRNA encoding a Milton ortholog was found strongly localized to the *Nasonia* oosome. Milton acts an adaptor that loads mitochondria onto microtubule motors for transport and localization within and between cells in *Drosophila* [78], and we propose that Nv-Milton may play a role in enriching mitochondria around the oosome and in the pole cells in the wasp, and perhaps other insect species that lack the specialization of long Osk isoform.

Another critical function for germ cells is the control of transposable elements, which is often dependent on Tudor domain-containing proteins. mRNAs for two Tudor domain proteins are present in the oosome, including *Nv-qin* and *Nv-tdrd7*. Neither of them is enriched in the polar granules or pole cells in *Drosophila*, but both have crucial roles during oogenesis to reduce the activity of transposable elements [47, 48, 79]. The presence of these additional Tudor domain encoding transcripts may suggest that either there is an increased activity of transposable elements in *Nasonia* that requires an earlier response, or perhaps other mechanisms are employed in *Drosophila* to combat transposon activity in the early PGCs. Further sampling of germ plasm of other insects should help to resolve these questions.

Germ cells are known to have a distinct metabolic profile from somatic cells, and this difference is related to their pluripotent stem cell-like properties, and to the requirements of their migratory properties [80, 81]. Potentially related to this, we have found that a transcript encoding an insulin homolog (*Nv-igf*) is localized to the oosome (Fig. 3C1). In addition, an mRNA encoding a putative organic cation transporter (*Nv-CG42269*) containing a Major Facilitator Superfamily (MFS) domain is strongly localized to the oosome. Such molecules are crucial for regulating cellular metabolism and signaling at multiple levels, by controlling the trafficking of many small organic molecules (including sugars) within and between cells [82, 83]. Lipid metabolism is also uniquely regulated in germ cells, and the identification of the *Nasonia* homolog of the Acyl-CoA reductase *waterproof* may reflect this [61]. Finally, we surprisingly found a transcript encoding a protein similar to endoglucanases found in several insect lineages (but absent from Diptera). The substrate and potential role for this enzyme in the *Nasonia* germline is not yet known.

In addition to providing insight into the conserved functions of germ cell components, we also found several molecules that do not have clear homology outside of the Hymenoptera, or in some cases outside of *Nasonia* and its closest relatives. This includes a novel RNA recognition domain containing protein whose function we analyzed in depth (*Nv-rrm* discussed in the following section). We also found that an mRNA encoding an ankyrin domain protein (*Nv-OoCLANK* (*LOC100679945*)) that belongs to a family of proteins that underwent a massive amplification within chalcid wasp lineage, and which appears to have entered the ancestral chalcid wasp by horizontal transfer [39]. Finally, a handful of transcripts have no identifiable domains or homologs. The functional relevance of these molecules will be an area of intense interest in the future.

### Discovery of factors required for maintaining oosome integrity

Early in our analysis, we chose a handful of transcripts for functional analysis. These were chosen based on a combination of criteria that included high enrichment in the oosome, novelty, and the potential (based on predicted molecular functions and roles of homologs in other systems) to give phenotypes that we could characterize with the current set of functional tools available in *Nasonia* (those that seemed most likely to affect integrity of the oosome, or the formation of the pole cells).

We focused on the effects of the RNAi on the oosome and pole cells, but we cannot exclude other developmental processes are being affected by the genes of interest. There are clearly disruptions in the morphology of the injected embryos (e.g., rougher blastoderm, less evenly spaced nuclei) compared to wild-type ones, but these disruptions were found in the eGFP RNAi embryos as well, suggesting that most of the deviations outside of the oosome and pole cells were due to the injection and/or fixation regimen required to analyze injected early embryos. Thus, we limited our discussion to phenotypes that were seen only in the RNAi for genes of interest, but that did not appear in the negative control, as they are highly likely to be phenotypes specifically caused by knockdown of the gene of interest.

Three of these (*Nv-bark*, *Nv-anillin*, and *Nv-rrm*) all gave the same unexpected phenotype, where oosome-like material was not coalesced into the typical spherical oosome structure, but rather was scattered in a film attached to the plasma membrane near the posterior pole. Eventually, these remnants of germ plasm-like material come into contact with syncytial nuclei when they migrate to the cortex. However, this remnant material is unable to induce the pole cell fate. It is important to reiterate that this phenotype is quite distinct from that seen for genes that have a core role in oosome assembly (*Nv-osk*, *Nv-tud*, *Nv-vas* [9, 28]). In these cases, there is no hint of the oosome, and mRNAs normally localized in the oosome are distributed in homogenous caps at the posterior pole, rather than as discrete clumps of material [9, 28].

The RNAi phenotypes of *Nv-bark*, *Nv-anillin*, and *Nv-rrm* suggest that these genes are involved in the coalescence and/or maintenance of the oosome into single entity within the central column of the cytoplasm in the embryo. The molecular bases of such functions are not completely clear at the moment for any of these three genes.

For example, Nv-Bark is a putative transmembrane protein and would be predicted to be targeted to the membrane. A hypothetical role for Nv-Bark could be that when this protein is targeted to the membrane at the posterior pole of the embryo, it causes the releases of oosome material from the plasma membrane, forcing the oosome material to enter the bulk cytoplasm where it concentrates into a large sphere subject to strong cytoplasmic flows that occur during the earliest cleavages. This model would also imply that interaction with the cortex prevents oosome material from coalescing, leading to the scattered clumps we observe in *Nv-bark* knockdowns. Mechanistically, this could be related to the ability of Bark to induce endocytosis [41], a process that is associated with proper anchoring of the germ plasm and the recruitment of specialized actin-binding proteins to the posterior pole in *Drosophila* [84].

An alternative hypothesis is that Nv-Bark produced in the oosome is not secreted, but is instead incorporated as an important structural component of the oosome. This could be consistent with the structure of the protein, which contains main protein-protein interaction domains of different types [40, 41]. Testing these hypotheses will require in-depth analysis of the subcellular localization of Nv-Bark during early embryogenesis, and proteomic analysis of binding partners of Nv-Bark.

Whatever the molecular function of *Nv-bark* in the oosome, we believe this molecule was recruited into a germ plasm role in the lineage leading to Nasonia. This is based on the conservation of Bark orthologs in Hemimetabola, which lack both germ plasm and pole cells, along with the good fit of the domain structure of Bark proteins with roles in mediating epithelial cell interactions. Further taxonomic sampling will be needed to determine whether this recruitment occurred early in the evolution of Holometabola, and was lost in the lineage leading to *Drosophila*, or whether it occurred within the Hymenopteran lineage, perhaps coinciding with the origin of the oosome type of germ plasm.

The role of *Nv-anillin* in maintaining the stability of the oosome was also surprising and the molecular basis of the phenotype will require further investigation. Anillin orthologs are well-known as actin-binding proteins involved in assembling the contractile ring required to separate cells in cytokinesis [45]. Anillin also plays a crucial role in the specialized cytokinesis of the *Drosophila* pole cells [46]. While these known functions might have suggested that oosome localization of *Nv-anillin* was related to an important role in the specialized polar bud formed in *Nasonia*, RNAi showed that this protein has an earlier role in oosome assembly/maintenance (Fig. 8A1). Similar to Nv-Bark, one possible function of Nv-Anillin is as a structural component of the oosome, which may or may not be related to its ability to bind actin and associated proteins. Alternatively, Nv-Anillin may act to release and/or repel the oosome from the cortex, as proposed above for Nv-Bark.

Alternatively, Anillin homologs have known functions that could be related to the release of germ plasm from the embryonic plasma membrane. In *Drosophila*, the germ plasm is tightly bound to the plasma membrane until the nuclei reach the posterior pole of the embryo. The centrosomes associated with these nuclei mediate detachment of the pole plasm from the cortex through interactions of the astral microtubules emanating from the centrosomes [85]. This is in contrast to *Nasonia*, where the oosome detaches from the cortex at about the same time as the zygotic nucleus begins its first division at the anterior pole. While nuclei are lacking at this time, numerous centrosomes are present, as they are provided maternally in a process characteristic of many Hymenopteran embryos [86]. Thus, Nv-Anillin could be relevant to a model where astral microtubules emanating from maternally provided centrosomes detach oosome material from the cortex, because Anillin homologs have been shown to mediate interactions between the actin cytoskeleton and cortical and subcortical microtubule arrays in multiple model systems [87, 88]. Again, in-depth examination of the subcellular localization and interactions of Nv-Anillin will be required to completely understand its role in maintaining the oosome.

Based on the presence of only RNA recognition motifs in the protein, we predict that Nv-Rrm will have one of two likely roles. One possibility is that it is involved in translational regulation of key regulators of oosome structure, presumably including *Nasonia* Anillin and *Nasonia* Bark. Alternatively, Nv-Rrm may be important in binding RNA and protein in order to maintain the structural integrity of the oosome. These possibilities are not mutually exclusive.

### Pole bud-specific factors

The knockdowns of *Nv-coronin* and *Nv-innexin1* had specific effects only on the formation of pole cells, while the oosome appeared to remain intact. Coronin is an actin-binding protein associated with the formation of highly concentrated networks of F-actin [58]. It seems likely that Nv-Coronin has an important role in organizing an actin cytoskeleton arrangement specialized for the formation and maintenance of the large polar bud that initiates pole cell formation.

The potential role of *Nv-innexin1* is somewhat more mysterious. Innexins are typically known as components of the gap junctions that are found in some tightly integrated epithelial tissues. Such junctions would not be expected of the motile pole cells. Interestingly, Innexin-7 (a paralogous protein with a similar structure to *Nasonia* Innexin1) in the beetle *Tribolium* has a novel role in cellularization of the syncytial blastoderm. A similar function for Nv-Innexin1 would explain the failure in pole cell formation we see after RNAi.

## Conclusion

This work has revealed numerous unexpected mRNAs that are localized to the germ plasm of the wasp *Nasonia.* The results will serve as the basis for hypotheses about the ancestral mechanisms used by insect embryos to accomplish conserved functions, in cases where diverged and lineage-specific mechanisms are used in *Drosophila melanogaster*. Examples include transporting and concentrating mitochondria to the germ plasm and repressing transcription in the newly formed PGCs*.* On the other hand, our results have identified numerous lineage-specific oosome components (e.g., *Nv-OoCLANK* and *Nv-rrm*) and conserved genes with unexpected functions in producing the unique characteristics of the oosome (such as the use of *Nv-bark* and *Nv-anillin* in assembling the oosome). We propose that these functions are novelties in the lineage of *Nasonia* and its parasitoid relatives where this unusual structure is found.

These hypotheses can be tested by deeper analyses of the functions of these molecules in *Nasonia*, broader sampling of germ plasm in other holometabolous insects, and characterizing the roles of homologs to germline genes in hemimetabolous insects that lack germ plasm. Such analyses will be important because the germ plasm is a uniquely powerful organelle that can rapidly drive naive nuclei into a highly specialized, yet functionally totipotent state. Understanding how and why such a fundamental substance changes, and is even lost, in the course of evolution will provide foundational insights into the mechanisms of cell fate determination and the interaction of subcellular organelles and their cellular milieu.

## Methods

### *Nasonia* rearing

Wasps were maintained in an incubator with temperature at 25 °C, relative humidity at 50%, and a 16:8 day to night light cycle. Pupae of the blow fly *Sarcopahga bullata* (Carolina Biological Supply Company) were used as hosts. After the *Nasonia* females emerged from the host, they were provided with filter paper soaked with 10% honey water for 2 days, after which they were transferred to egg-laying chambers for embryo collection. Wasps were used for egg lays for a maximum of 3 days, and no variation in egg quality have been detected under these routine conditions in our hands.

### RNAseq sample preparation

In order to identify the maternally deposited mRNAs in the posterior half of *Nasonia* embryos, we collected and bisected the pre-blastoderm-stage embryos (0–2 h after egg lay at 25 °C) to detect the differential mRNA levels between the anterior and posterior halves of the embryos, using a device and experimental procedure derived from experiments in *Drosophila* [32].

The embryos were affixed to a pre-chilled slide with heptane glue, aligned with their anterior poles to the right. They were then covered with a thin layer of halocarbon oil 700 (Sigma) The slide was transferred to a pre-chilled “guillotine” and placed on dry ice. After the halocarbon oil 700 was solidified, the apparatus was placed on pre-chilled aluminum block to maintain cold temperature. Embryos were aligned with a blade slot on the guillotine, and a fresh razor was pressed down to the slide to bisect the embryos. After the embryos were cut, the anterior and posterior halves of the embryos were immediately collected in strips of solidified halocarbon oil and transferred into 1.5-mL non-stick RNase-Free microfuge tubes (Ambion) with a pre-chilled probe. Samples were immediately homogenized in TRIzol (Ambion 15596018). After phase separation with the addition of chloroform, RNA was precipitated with an equal volume of isopropanol. The pellet was then washed in 70% ethanol and re-suspended in RNAse-free water.

Total RNA was isolated from these six (three anterior and three posterior) samples for library preparation. Due to some difficulties with our first attempt (see RNAseq quality control and analysis, below), the library production procedure was performed twice with a few alterations at multiple steps of the protocol. For experiment 1, 1 μg of total RNA was collected for each sample and used for library preparation. Collection of this amount of RNA for each sample required three rounds of alignment and bisections per replicate. For experiment 2, we took an approach similar to work with low quantities of RNA from thin sections of *Drosophila* embryos [89]. One hundred nanograms of total RNA from *Nasonia* embryo fragments was spiked into 900 ng of RNA from *Melittobia digitata* [39] that acted as a carrier. Enough RNA from *Nasonia* was obtained from a single bisection for each replicate. For experiment 1, library preparation was performed using the PrepX mRNA kit (Takara). For experiment 2, libraries were prepared using the NEBNext Ultra Directional RNA Library Prep Kit for Illumina (NEB #E7420) in conjunction with NEBNext Poly(A) mRNA Magnetic Isolation Module (NEB #E7490). For both experiments, libraries were validated and quantified before being pooled and sequenced on an Illumina HiSeq 2000 sequencer with a 100-bp paired-end protocol. Sequence files for experiment 1 are available in NCBI under Biosamples SAMN10624419-SAMN10624430, and those for experiment 2 are under SAMN09762220-SAMN09762225. All raw sequencing data is available and collected under Bioproject PRJNA484241 [90].

### RNAseq quality control and data analysis

The quality of the sequencing data was determined using FastQC software [91–93]. This revealed many difficulties with experiment 1, including the following: (1) many sequenced fragments were significantly smaller than the expected 200–300 bp, (2) a large number of duplicate reads, suggesting over-amplification of the library, and (3) a major artifact in the flow cell affecting all second strand reads (see FastQC results in [92]). When all three replicates were used in our analysis pipeline ([94], analysis 1), very few transcripts met the significance threshold set in Cuffdiff ([95], analysis 1). However, when replicate 1 was removed from the analysis ([94], analysis 2), many more transcripts were found to be differentially enriched ( [95], analysis 2), suggesting that replicate 1 was compromised in some way that obscured differential enrichment between the fragments.

Quality control results of the experiment 2 sequences [96] were similar to those obtained in our previous RNAseq experiments that were successful [39, 43], so we proceeded to analyses of differential enrichment between the anterior and posterior halves of the embryos.

Additional quality control was performed with CummeRbund, which showed that inter-replicate variability was low in experiment 2 (Additional file 6: Figure S5) compared to other high-quality RNAseq experiments given as examples in the CummeRbund manual (http://compbio.mit.edu/cummeRbund/manual_2_0.html). The sequences were processed by Cufflinks package for differential expression detection, using multiple variations on the default parameters (job files that show the different parameters used in the mapping and quantifying stages are given in the file in [94, 96]. The names of the analyses in [94, 96] correspond to the names of the tabs that give the Cuffdiff results in [95, 97]).

For these analyses, several parameters were varied in the mapping, quantification, and differential expression steps (see [96] and “Methods”). Our previous experience had shown that varying these parameters gave slightly, but meaningfully, different results [43]. Two different annotations of the *Nasonia* genome were used to aid in assembly and quantification of the reads: OGS 2.0 ([96], analyses 1–3) and annotation 2.1 version 102 from NCBI ([96], analyses 4–7). We also varied mapping conditions, by allowing ([96], analyses 1, 2, 4, and 6) or preventing ([96], analyses 3 and 5) the prediction of novel junctions during the genome mapping step in Tophat2. One analysis was mapped only to the transcriptome, without any genome mapping ([96], analysis 7).

Each analysis gave slightly different results, with each differing from the others by only a few different genes being identified as significantly enriched at one pole ([97], see “compiled 2.1transcriptome” tab). However, all of the significant loci were found to be significant in at least two analyses, which gave us confidence that they were worthy of consideration.

In Cuffdiff, default values were used for the false discovery rate (FDR, 0.05), type of normalization (FPKM), dispersion model (negative binomial), and significance cutoff (*q* < 0.05). FDR was used to convert the raw *p* value into a *q* value. Detailed description of the statistical methods used to generate the test statistic and significance values can be found at [98].

Homologs of the differentially expressed genes were identified using reciprocal BLAST and corroborated with orthoDB [99].

In the end, experiment 1 is described here only because, despite its many problems, a handful of confirmed posteriorly localized factors were identified in this analysis that were not calculated to be statistically significantly enriched in the second experiment, including the *Nasonia* homolog of *coronin* (*Nv-coronin*). Because we began the functional analyses before the second set of sequencing was analyzed, we made significant progress in working with *Nv-coronin* before the second analysis gave a “NOTEST” result for this gene, meaning that no statistical test was performed by Cuffdiff to determine significant enrichment. Such a result is normally returned when too few alignments are present for a particular locus, according to Cuffdiff [98].

Rather than discarding *Nv-coronin*, (and the other genes found only in experiment 1) and focusing only on results gained in experiment 2, we decided it was more in the spirit of our approach to include all of the informative results from both sets of experiments. In short, our goal was to identify as many posteriorly localized factors as possible, in order to better understand the structure and function of the oosome. Thus, we considered the output of the statistical tests and modeling contained in the Cufflinks/Stringtie and Cuffdiff algorithm as means to identify candidates for further confirmation, rather than as ends in themselves.

### Assessment of effects of Melittobia carrier RNA

*Melittobia* is a distant (96 million yeas diverged) relative of *Nasonia*, and, for the vast majority of cases, the paired 100-bp reads are sufficient to differentiate the species of origin. Therefore, in the analyses presented here, all Tophat2 mapping was done from the raw mixed reads to the *N. vitripennis* genome, resulting in the expected ~ 10% mapping rate (given the 9:1 ratio of *Melittobia* to *Nasonia* RNA used to create the libraries). To further explore the potential issue, we took the reads that mapped to the *Nasonia* genome and mapped them to the *Melittobia* genome (commands given in [100]). We found about 5% of the 8 million reads that mapped to the *Nasonia* genome (of the replicate [2] we examined in detail) also mapped to the *Melittobia* genome. We found that these reads map in total to ~ 222,000 bp on the *Nasonia* genome, which is less than 0.1% of the 300-Mb *Nasonia* genome assembly, and less than 1% of the total length of annotated exons (~ 78 Mb). The relatively high rate of individual read mapping is likely due to the high expression level of many of the nearly identical genes, which include several that encode ribosomal proteins. None of the transcripts with significant mapping to both Nasonia and Melittobia had significant signal of localization in the first experiment (where Melittobia RNA was not used).

To determine whether reads that map to both genomes affected our results, we mapped the raw reads first to the *Melittobia* genome, converted the unmapped reads back to FASTQ format, and then mapped those reads that did not map to *Melittobia* to the *Nasonia* genome (commands for this analysis are found at [100]). The results were nearly identical to previous analyses of the second dataset, and all of the confirmed localized transcripts were found [101]. On the other hand, we found three transcripts where the *Melittobia* carrier seems to have led to a false positive result in Cuffdiff. These were the transcripts *LOC100122199*, *LOC100115727*, and *LOC100678565.* Mapping to *Melittobia* first either removed detectable sequence completely or removed significant differential expression between the conditions. Note that these did not show localized expression with in situ hybridization (Additional file 2: Figure S2).

### Probes and dsRNAs

Probes and dsRNAs for the chosen genes were generated by the protocol described in [28]. Primers for generating these templates are provided in [102]. Alkaline phosphatase in situ hybridization was performed by the protocol described in [103]. In situ hybridization results of transcripts tested that showed no enrichment or localization are given in Additional file 2: Figure S2.

### Embryonic RNA interference (eRNAi)

In order to perform eRNAi on the early *Nasonia* embryos to study the germline candidate genes’ functions, we created the following workflow:

Around 30 pre-blastoderm-stage embryos (0–1 h after egg lay of females 1–3 days past eclosion at 25 °C) were collected and quickly aligned vertically on the heptane glue-coated 18 mm × 18 mm coverslip. This coverslip was transferred and anchored on the pre-chilled slide by applying a thin layer of water. The slide was then put in a petri dish with a layer of desiccant (Drierite with indicator, 8 mesh, ACROS Organics) pre-chilled at 4 °C. The embryos were dehydrated in the desiccant at 4 °C for 45 min. After dehydration, the embryos were covered with a layer of halocarbon oil 700 and were ready for microinjection.

The dsRNAs were dissolved in Nuclease-Free Water (Ambion) at the concentration of 1 μg/μL and loaded into the Femtotips II Microinjection Capillary (Cat. No. 930000043, Eppendorf). The constant pressure was set at 500 hpa and the injection pressure was set initially at 250 hpa with periodic adjustment as the needle changed over the course of injection. The process of injection was performed at room temperature and needed to be done as soon as possible to minimize the amount of development that could occur prior to the dsRNA acting to knock down the target transcript. After injection, the slide was transferred into a paper towel-moisturized petri dish pre-warmed at 28 °C to incubate the injected embryos for specific developmental stages. The embryogenesis of these embryos was stopped at the pre-blastoderm stage (before the budding), beginning of the blastoderm stage (during budding), and later in the blastoderm stage (pole cells formed and/or after pole cell divisions). To stop the development, the coverslip was put into the heptane to wash off the halocarbon oil 700 for 3 min, and then transferred into the 37% formaldehyde-saturated heptane for 2–5 h fixation in the dark with the embryos facing up.

After fixation, the coverslip was taken out of the fixative and flipped upside down to gently press the embryos on a double-sided tape that was taped on a petri dish, so that all the embryos can be anchored on the tape for dissection. About 15 mL PBS with 1% Tween was poured over the affixed embryos, and a hypodermic needle (BD PrecisionGlide Needle, 30 G × 1) was used to remove the eggshells from the embryos. The dissected embryos were then transferred by pipette into the 1.5-mL non-stick RNase-Free microfuge tubes. The embryos were immediately dehydrated by 100% methanol and stored at − 20 °C.

### Evaluating embryos for data collection

As a negative control, we injected dsRNA against eGFP to test whether the physical injection and/or dsRNA would affect the structure of the oosome and formation of the pole cells non-specifically.

In some cases, non-specific injection damage was severe, leading to non-specific disrupted development and eventual death of the embryo. We set a stringent criterion for collecting embryos for later analysis by removing those where yolk leakage exceeded more than about 10% of the embryo size (although all embryos with this amount of damage showed normal germ plasm and germline development). We also excluded embryos that showed major morphological changes as compared to uninjected embryos.

After removing the embryos with obvious major damage both after injections, and when performing eggshell dissection after the fixation, we were left with about 85% embryos with viable embryogenesis by the time of imaging after in situ hybridization. The same criteria were also applied to the experimental knockdowns where the percentages were roughly the same as in the negative control. When determining the phenotypes for the five genes, we considered the disruptions of developmental events to be potential effects of a knockdown when they were only specific to the knockdown and were never observed in the negative control.

We performed the same procedures when injecting and collecting the embryos as described for the negative control. The penetrance given by the embryonic RNAi (eRNAi) knockdowns is higher than the pRNAi knockdown, ranging from 20 to 39% across experiments.

Before performing fluorescent in situ hybridization (FISH) on those eRNAi knockdown embryos, they were rehydrated with a series of methanol/PBT washes (75%, 50%, 25%). The protocol for FISH was adapted from [28]. A detailed protocol is available on request.

### qPCR validation of embryonic RNAi

Egg lays and injection procedures were identical to those described above. Thirty eggs were injected per sample subjected to qPCR. After injection, we aged the pre-blastoderm embryos for 3 h at 25 °C and subsequently extracted RNA using TRI Reagent (Sigma Aldrich cat. No T9424). To ensure we had high-quality RNA, we quantified and assessed the quality using a 2100 Agilent Bioanalyzer. Reverse transcription was performed with the Protoscript First Strand cDNA synthesis kit (NEB 6300I) and anchored oligo-dT primers. Three biological replicates (independent injections of dsRNA) were performed for each gene, and each sample was subject to three technical replicates. Nv-rp49 was used as a control to normalize the gene-specific values. Values for comparison were calculated with the delta-delta C(T) method [104], and significance was calculated with *t*-tests [105]. Data and calculations are available at [105]. Primers used specifically for qPCR are given below.

**Table:** 

qPCR primer name	Sequence
Nv-anillin-RT-F	CGTCACGTAGAGCCTGAGAC
Nv-anillin-RT-R	TCAGCTTGTACAACAGGCGT
Nv-innexin1-RT-F	CCGAGACGTATATGCTGCGT
Nv-innexin1-RT-R	AATCGGCTCGAGTCATGTGG
Nv-rrm-RT-F	ATTCGCTGAAAGTCGCCAGA
Nv-rrm-RT-R	GTGCTCTCGTTGGTGTTTTGA
Nv-bark-RT-F	GTACGATTGTCGCGGGTACT
Nv-bark-RT-R	TTTCCAATTGAAGTGATCTGTTGAT
Nv-coronin-RT-F	GGTATACACTGCCCGCATCT
Nv-coronin-RT-R	AAAGAGATGACTCGACCGGC
Nv-rp49-RT-F	GTGTACAGGCCGAAAATCGT
Nv-rp49-RT-R	GCTTCCTCCAGTTACGCTTG

## Additional files

Additional file 1: **Figure S1.** Expression of genes with localized enrichment not shown in main text. All embryos are in pre-blastoderm stage, ~0-2 hours old, with posterior side to the right and dorsal side on the top. Corresponding expression level data for these transcripts can be found in [33]. Scale bar indicates 50 microns. (TIFF 15021 kb)

Additional file 2: **Figure S2.** Genes without visually detectable enrichment by in situ hybridization. All embryos are in pre-blastoderm stage, with posterior side to the right and dorsal side on the top. Detailed information about the genes can be found in [33]. Scale bar indicates 50 microns. (TIFF 11271 kb)

Additional file 3: **Table S1.** Posterior-enriched genes in Nasonia. The posterior-enriched genes identified in the RNA-seq experiment and validated by in-situ hybridization are sorted into 5 of the following functional categories: cellular transport/ localization (★), cytoskeletal structure/cell organization(△), transcriptional/translational regulation(◇), metabolism(☐) and unknown(•).(XLSX 43 kb)

Additional file 4: **Figure S3.** pRNAi phenotypes in different embryonic stages. (A) *Nv-innexin1* pRNAi phenotype in blastoderm stage. (B-C) *Nv-rrm* pRNAi phenotypes in early blastoderm stage (B) and mid-blastoderm stage (C). (D-F) *Nv-coronin* pRNAi phenotypes in early blastoderm stage (D), mid-blastoderm stage (E) and post-gastrulation stage (F). Embryos in A, B, D are obtained from alkaline phosphatase in situ hybridization detection using probe against *Nv-bark*. Nv-bark expression was detected using fluorescent tyramide detection in panels C, E, and F. All embryos are aligned with posterior side to the right and dorsal side on the top. Scale bar indicates 50 microns. (TIFF 3946 kb)

Additional file 5: **Figure S4.** qPCR quantification of knockdown in eRNAi experiments. Gray bars represent delta (Ct) values comparing the levels of mRNA from the gene of interest to standard *Nv-rp49* in embryos injected with eGFP dsRNA (ctrl). Black bars represent the same comparison in embryos that were injected with dsRNA against the indicated gene. Fold change was calculated using the differences between the Ct values of control and gene specific cases. *p*-values were calculated through t-tests. Three independent biological replicate experiments were used to assess the knockdown for each gene, and within these three technical replicates were used for each sample. Each pool of RNA was produced from ~30 injected embryos. Data and calculations are available at [105]. (TIFF 9096 kb)

Additional file 6: **Figure S5.** Distance matrix of replicates for Experiment 2. Shading indicates magnitude of the pairwise Jensen–Shannon (JS) distance between all replicates and conditions. The similarity in magnitude of all of the values indicates there are no strong outliers among the data. (JPG 250 kb)

## Data Availability

All data generated and analyzed during this study are included in this published article, in its supplementary information files, or in cited online repositories [33, 92–97, 100–102, 105]. Sequencing results can be found in the NCBI BioSamples database under accession numbers SAMN10624419-SAMN10624430 and SAMN09762220-SAMN09762225 [90].
