# Supplementary figures and images for: Transcriptomic and functional analysis of the oosome, a unique form of germ plasm in the wasp Nasonia vitripennis
Source: BMC Biol. 2019 Oct 10;17:78. doi: 10.1186/s12915-019-0696-7 (PMC6785909; doi:10.1186/s12915-019-0696-7)

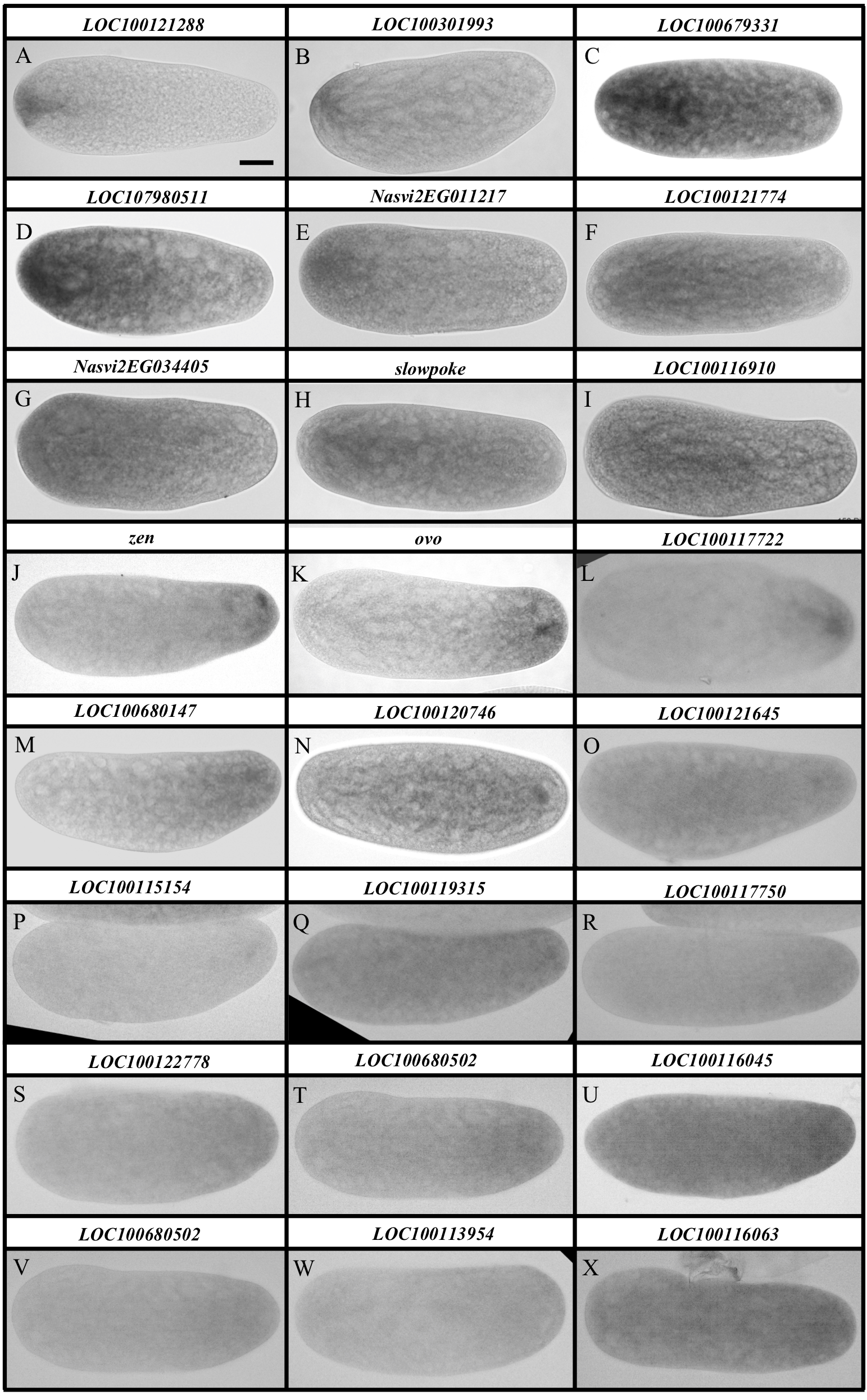

Supplement: Supplementary file 1 — Figure S1. Expression of genes with localized enrichment not shown in main text. All embryos are in pre-blastoderm stage, ~0-2 hours old, with posterior side to the right and dorsal side on the top. Corresponding expression level data for these transcripts can be found in [33]. Scale bar indicates 50 microns. (TIFF 15021 kb) [file 12915_2019_696_MOESM1_ESM.tiff]

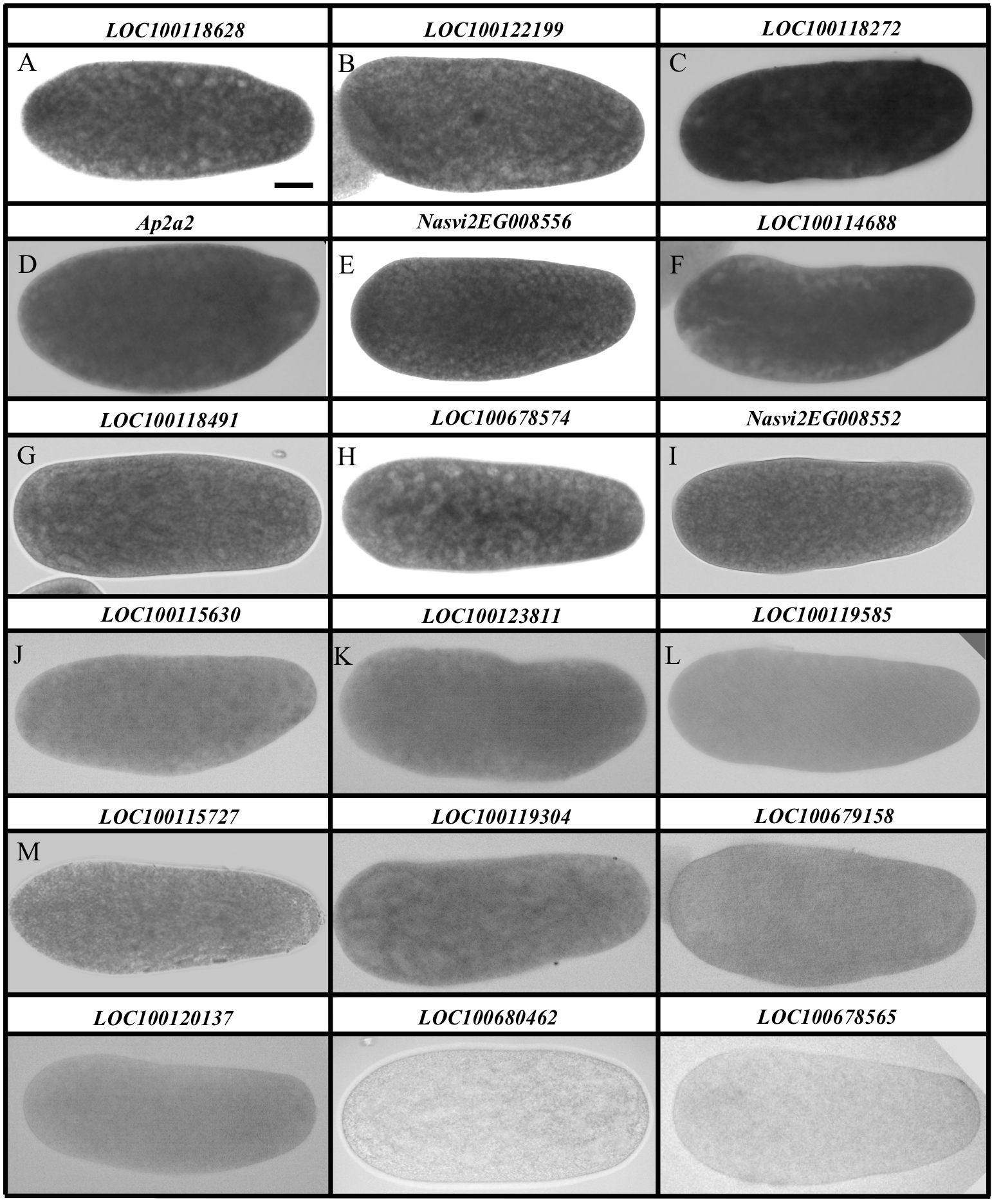

Supplement: Supplementary file 2 — Figure S2. Genes without visually detectable enrichment by in situ hybridization. All embryos are in pre-blastoderm stage, with posterior side to the right and dorsal side on the top. Detailed information about the genes can be found in [33]. Scale bar indicates 50 microns. (TIFF 11271 kb) [file 12915_2019_696_MOESM2_ESM.tiff]

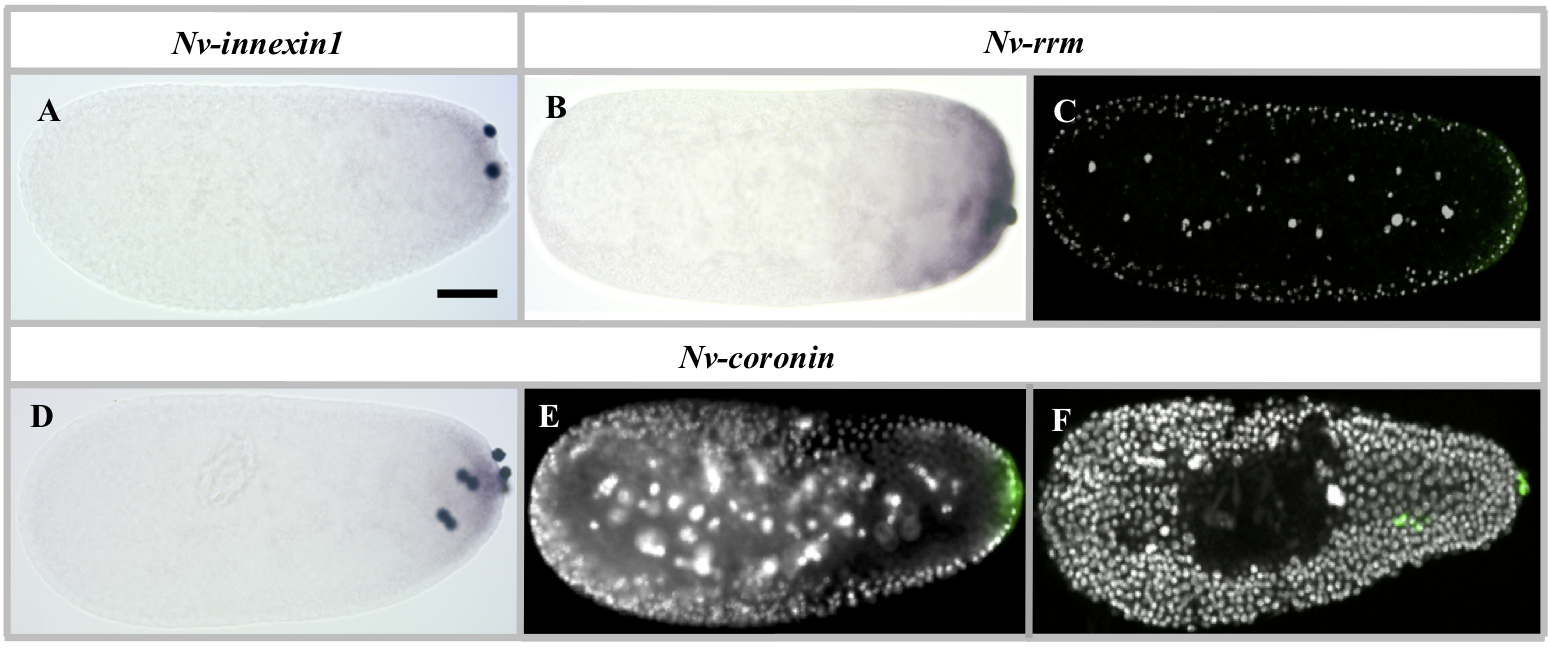

Supplement: Supplementary file 4 — Figure S3. pRNAi phenotypes in different embryonic stages. (A) Nv-innexin1 pRNAi phenotype in blastoderm stage. (B-C) Nv-rrm pRNAi phenotypes in early blastoderm stage (B) and mid-blastoderm stage (C). (D-F) Nv-coronin pRNAi phenotypes in early blastoderm stage (D), mid-blastoderm stage (E) and post-gastrulation stage (F). Embryos in A, B, D are obtained from alkaline phosphatase in situ hybridization detection using probe against Nv-bark. Nv-bark expression was detected using fluorescent tyramide detection in panels C, E, and F. All embryos are aligned with posterior side to the right and dorsal side on the top. Scale bar indicates 50 microns. (TIFF 3946 kb) [file 12915_2019_696_MOESM4_ESM.tiff]

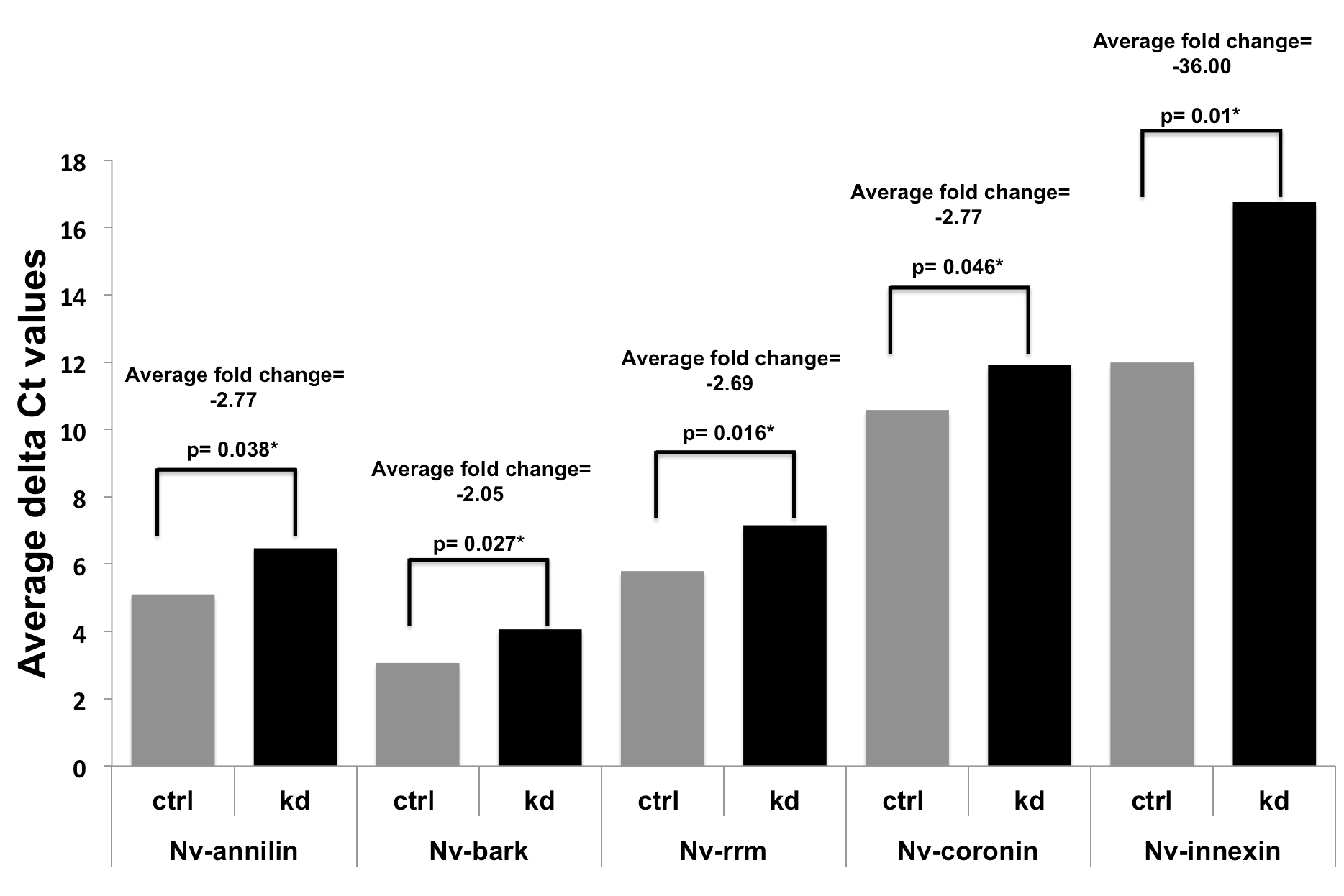

Supplement: Supplementary file 5 — Figure S4. qPCR quantification of knockdown in eRNAi experiments. Gray bars represent delta (Ct) values comparing the levels of mRNA from the gene of interest to standard Nv-rp49 in embryos injected with eGFP dsRNA (ctrl). Black bars represent the same comparison in embryos that were injected with dsRNA against the indicated gene. Fold change was calculated using the differences between the Ct values of control and gene specific cases. p-values were calculated through t-tests. Three independent biological replicate experiments were used to assess the knockdown for each gene, and within these three technical replicates were used for each sample. Each pool of RNA was produced from ~30 injected embryos. Data and calculations are available at [105]. (TIFF 9096 kb) [file 12915_2019_696_MOESM5_ESM.tiff]

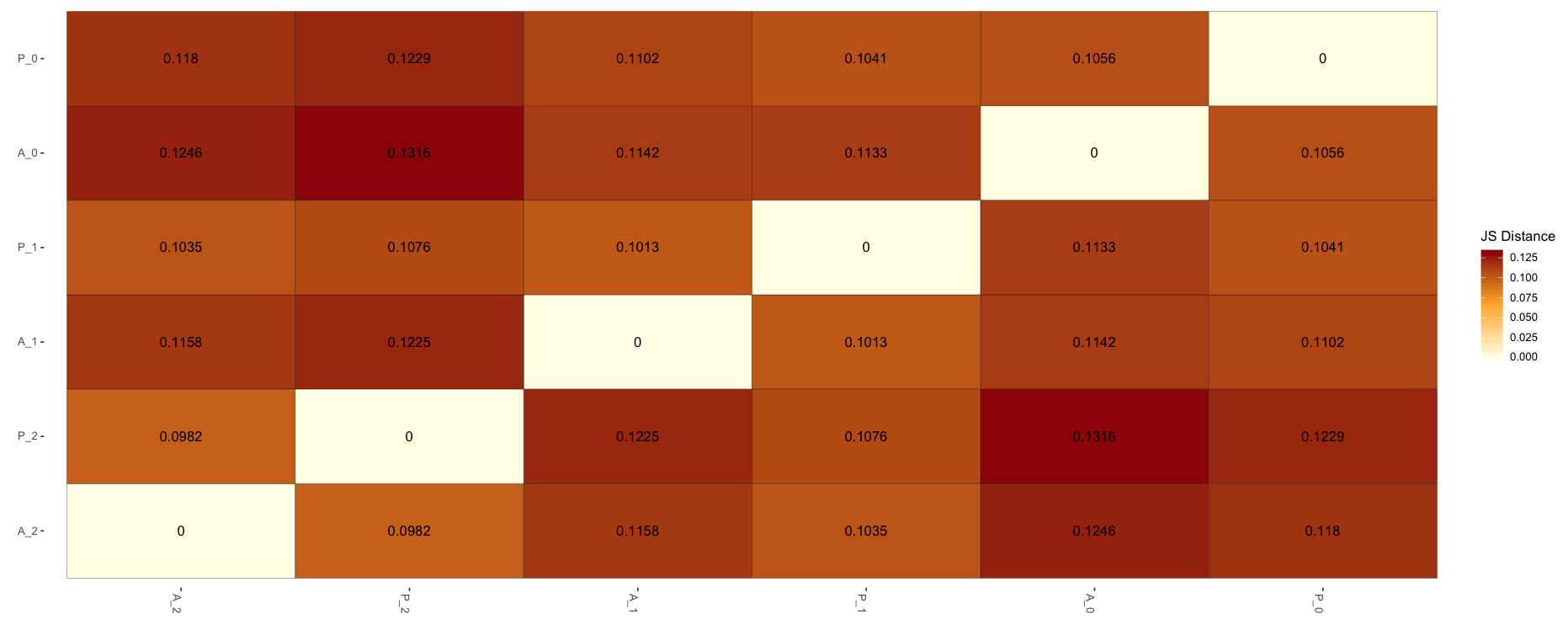

Supplement: Supplementary file 6 — Figure S5. Distance matrix of replicates for Experiment 2. Shading indicates magnitude of the pairwise Jensen–Shannon (JS) distance between all replicates and conditions. The similarity in magnitude of all of the values indicates there are no strong outliers among the data. (JPG 250 kb) [file 12915_2019_696_MOESM6_ESM.jpg]
